# Extracellular matrix rigidity modulates physical properties of subcapsular sinus macrophage-B cell immune synapses

**DOI:** 10.1016/j.bpj.2023.10.010

**Published:** 2023-10-14

**Authors:** Maro Iliopoulou, Anna T. Bajur, Hannah C.W. McArthur, Michael Gabai, Carl Coyle, Favour Ajao, Robert Köchl, Andrew P. Cope, Katelyn M. Spillane

**Affiliations:** 1 Department of Physics, King’s College London, London, United Kingdom; 2 Randall Centre for Cell & Molecular Biophysics, King’s College London, London, United Kingdom; 3 Centre for Inflammation Biology and Cancer Immunology, King’s College London, London, United Kingdom; 4 Peter Gorer Department of Immunobiology, King’s College London, London, United Kingdom

## Abstract

Subcapsular sinus macrophages (SSMs) play a key role in immune defense by forming immunological barriers that control the transport of antigens from lymph into lymph node follicles. SSMs participate in antibody responses by presenting antigens directly to naive B cells and by supplying antigens to follicular dendritic cells to propagate germinal center reactions. Despite the prominent roles that SSMs play during immune responses, little is known about their cell biology because they are technically challenging to isolate and study in vitro. Here, we used multicolor fluorescence microscopy to identify lymph node-derived SSMs in culture. We focused on the role of SSMs as antigen-presenting cells, and found that their actin cytoskeleton regulates the spatial organization and mobility of multivalent antigens (immune complexes [ICs]) displayed on the cell surface. Moreover, we determined that SSMs are mechanosensitive cells that respond to changes in extracellular matrix rigidity by altering the architecture of the actin cytoskeleton, leading to changes in cell morphology, membrane topography, and IC mobility. Changes to extracellular matrix rigidity also modulate actin remodeling by both SSMs and B cells when they form an immune synapse. This alters synapse duration but not IC internalization nor NF-κB activation in the B cell. Taken together, our data reveal that the mechanical microenvironment may influence B cell responses by modulating physical characteristics of antigen presentation by SSMs.

## Significance

Subcapsular sinus macrophages (SSMs) capture lymph-borne antigens and present them to naive B cells, initiating antibody responses. SSMs maintain a fixed position in the lymph node by adhering to an extracellular matrix (ECM) that changes in stiffness during an immune response. In this paper, we show that ECM-SSM tension alters physical properties of immune complex (IC) presentation by SSMs to B cells. Alterations in ECM rigidity impact actin remodeling by SSMs and B cells in the immune synapse. These changes impact synapse duration, but do not affect the ability of B cells to internalize ICs or activate NF-κB transcription. Our data suggest that ECM mechanics may help orchestrate antibody responses by altering physical properties of SSM-B cell immune synapses.

## Introduction

Antigen-triggered B cell activation is the first step toward the production of antibodies and the establishment of immunological memory (1). B cells are activated through direct contacts with antigen-presenting cells (APCs), which display foreign antigens on their surfaces for B cells to sample through their B cell receptors (BCRs) (2). Specific binding interactions between the BCR and antigen prompt the B cell and APC to form a structured cell-cell interface called the immune synapse (3). Molecular interactions in the synapse induce BCR signaling, which stimulates transcriptional activation leading to B cell proliferation and differentiation (4), and BCR internalization of antigen (5), which is required for B cells to obtain and present antigenic peptides to recruit T cell help (6). The strength of BCR signaling, and the amount of antigen internalized, generally correlate with the affinity of the BCR-antigen interaction (7,8). This mechanism promotes B cell discrimination of antigens and drives the affinity maturation of antibodies (9).

To dissect the molecular requirements of B cell activation, researchers have stimulated B cells with artificial substrates that mimic APCs, such as planar lipid bilayers and hydrogels. These materials mimic essential features of APC membranes while offering a more controllable environment, which has been essential for establishing how immobilized antigens stimulate a B cell response. Experiments using these antigen presentation platforms have revealed that BCRs exert mechanical forces on surface-anchored antigens to fine-tune signaling responses (10) and antigen discrimination (11). Further research has shown that BCR-generated forces enable B cells to sense physical attributes of antigen presentation, including antigen spacing (12), antigen mobility (13), substrate stiffness (14,15), and substrate topography (16). These findings were later confirmed to be relevant for B cell contacts with live APCs, which use their actin cytoskeletons to modulate membrane stiffness and ligand mobility to regulate lymphocyte activation (15, 17).

**Figure 1:**
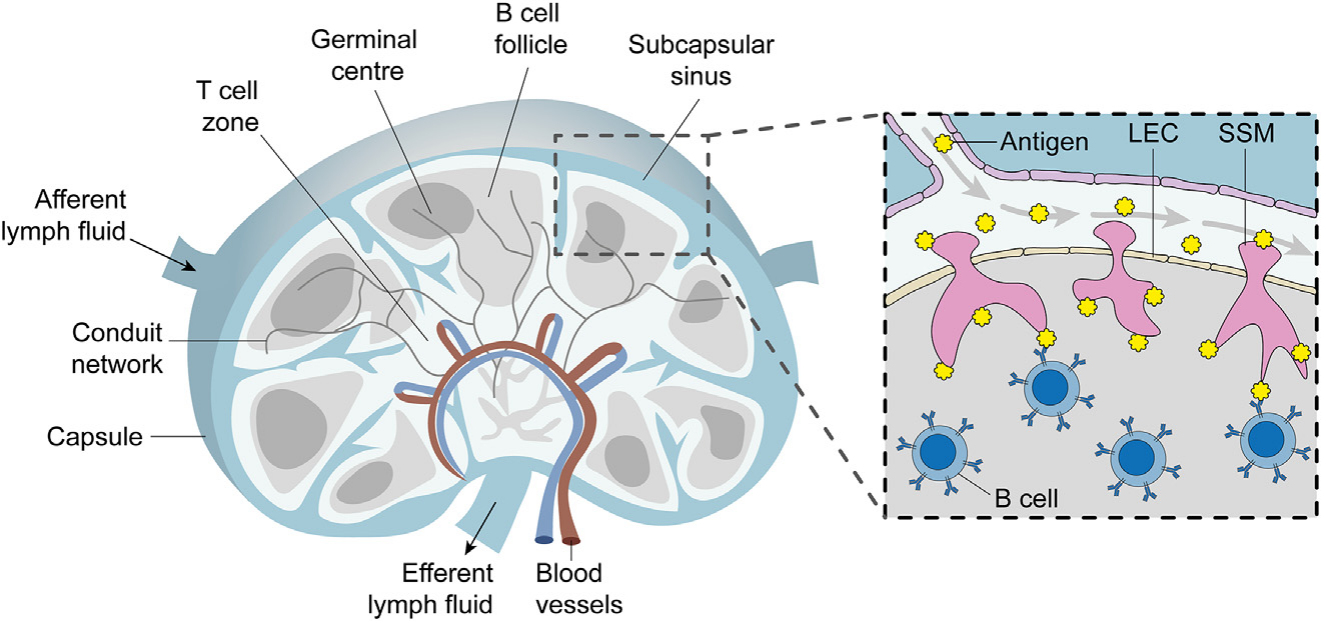
Schematic view of the lymph node. Antigens suspended in lymphatic fluid are swept over subcapsular sinus macrophages (SSMs) that interdigitate a layer of lymphatic endothelial cells (LECs) to protrude into the B cell follicle. SSMs capture antigens from the lymph and transfer them into the follicle to present to B cells. To see this figure in color, go online.

In vivo, the activation of naive B cells relies on the arrival of exogenous antigens via afferent lymphatic vessels, which propel lymph-borne antigens over a layer of CD169^+^ macrophages that line the subcapsular sinus floor (Fig. 1). Subcapsular sinus macrophages (SSMs) have a distinct morphology that underpins their dual roles as immunological barriers and APCs. They interdigitate a layer of lymphatic endothelial cells (18), protruding a “head” into the lymph to capture antigens (19,20,21,22) and extending long “tail” processes deep into the follicle to present antigens directly to B cells (21) and to transfer them to follicular dendritic cells (FDCs) for long-term retention (23,24). SSMs thus play a key role in naive B cell activation by acting as APCs, and in B cell affinity maturation by controlling antigen deposition on FDCs, which supply antigens to B cells during germinal center reactions.

SSMs maintain their position in the lymph node, in part, through adhesion to an extracellular matrix (ECM) that is enriched with collagen I (25,26). In response to immune challenge, the ECM network remodels to accommodate rapid lymph node expansion, resulting in global changes to network tension (27,28). Many cell types modulate their morphology and interactions with other cells in response to mechanical forces transmitted and detected through integrin-ECM bonds (29). However, the downstream impacts of ECM mechanical changes on SSMs—especially in the context of antigen presentation to B cells—are unknown. A major limitation to understanding SSM (mechano)biology is that the cells are difficult to procure; they are limited in number, highly sensitive to manipulation, and prone to die during isolation from lymph node tissue. We consequently do not understand how naive B cells are activated by these APCs, which are central to the adaptive immune response.

In this study, we developed an efficient method to enrich SSMs from mouse lymph nodes for single-cell studies in vitro. Using quantitative imaging and biophysical approaches, we characterized their presentation of antigens, response to the mechanical environment, and interactions with naive B cells. We demonstrated that the SSM actin cytoskeleton regulated both the spatial organization and mobility of antigens presented on the SSM surface. These physical aspects of antigen presentation were not fixed features of SSMs, but rather dynamic properties that adapted in response to changes in ECM composition and stiffness. Furthermore, alterations in ECM rigidity manifested as changes in actin remodeling within both SSMs and B cells in the immune synapse. These changes impacted the duration of the immune synapse, but they did not impede the ability of B cells to acquire immune complexes (ICs) or activate NF-κB transcription.

## Materials and methods

### Mice

C57BL/6 mice were used as a source of SSMs and naive B cells. B1-8^flox/flox^ Igκ^Ctm1Cgn/tm1Cgn^ mice on a C57BL/6 background (B1-8 mice) were used as a source of naive B cells specific for the NIP and NP haptens. Mice were housed in the King’s College London Biological Services Unit under specific pathogen-free conditions. All mice were age 6–16 weeks, and both males and females were used. Mice were maintained and treated following guidelines set by the UK Home Office and the King’s College London Ethical Review Panel.

### SSM isolation and culture for imaging experiments

Lymph nodes (superficial and deep cervical, brachial, axillary, mesenteric, and inguinal) were harvested from five C57BL/6 mice and teased apart using 25G needles in a 35 mm petri dish containing 1 mL DMEM medium. Another 1 mL DMEM medium containing 0.625 mg/mL DNase1 (Roche, Basel, CH) and 0.26 U Liberase DH (Sigma-Aldrich, St. Louis, MO) was added to the cells and incubated for 45 min at 37°C. Released cells were collected, and the tissue was digested a second time with fresh reagents. The cells were pooled and passed through a 70-*μ*m cell strainer and pelleted by centrifugation for 7 min at 300 × *g*. The cell pellet was resuspended in 200 *μ*L ice-cold DMEM and incubated sequentially on ice with purified rat anti-mouse FDC-M1 (BD Biosciences, Franklin Lakes, NJ) or rat IgG2c, κ isotype control antibody (1.6 *μ*g antibody per 2 × 10^7^ cells) for 1 h, 1 *μ*g biotinylated mouse anti-rat Ig, κ light chain (BD Biosciences, Franklin Lakes, NJ) antibody for 40 min, and 50 *μ*L anti-biotin microbeads (Miltenyi Biotec, Bergisch Gladbach, DE) for 20 min. The cells were pelleted (5 min at 300 × *g*) and resuspended in full DMEM (DMEM supplemented with 10% heat-inactivated fetal calf serum, 20 mM HEPES, 0.2 mM MEM nonessential amino acids, and penicillin-streptomycin-glutamine; all from Gibco, Thermo Fisher Scientific, Waltham, MA) between each incubation. Following a final wash and resuspension of the cell pellet in MACS buffer (PBS [pH 7.4], 1 mM EDTA, 0.5% BSA), cells were isolated by positive selection using an LS Column and MidiMACS Separator (Miltenyi Biotec). Cells were resuspended in full DMEM and plated onto collagen I-coated glass or polyacrylamide gels in FluoroDish cell culture dishes (10-mm well; World Precision Instruments, Sarasota, FL) at a density of 3 × 10^5^ cells per dish. Cells were cultured at 37°C with 5% CO_2_. The media was exchanged at 48 h to remove dead lymphocytes and cell debris. Cells were imaged on days 5–7.

Flow cytometric analysis showed that 13% of cells in the positive fraction were SSMs; most remaining cells were CD11b^–^ CD11c^–^ lymphoid cells (Fig. S1). In culture, lymphocytes adhered to SSMs for several days following isolation (Fig. S2), which impeded analysis of SSM membrane morphology, actin structures, and IC localization. We therefore imaged cells on days 5–7 post-isolation, at which time the lymphoid cells had died and could be washed away.

For actin perturbation experiments, cells were exchanged into warm Hanks’ balanced salt solution (HBBS) (+Ca^2+^, +Mg^2+^; Gibco, Thermo Fisher Scientific, Waltham, MA) supplemented with 0.01% BSA (HBSS 0.01% BSA) and mycalolide B, latrunculin B, jasplakinolide, CK-666, SMIFH2, Y-27632, or blebbistatin at final concentrations listed in Table S2. Control cells received an equivalent amount of DMSO diluted in warm HBSS 0.01% BSA.

### B cell isolation and culture

Primary splenocytes were obtained by passing the spleen through a 70-*μ*m cell strainer and lysing red blood cells using RBC lysis buffer (eBioscience, San Diego, CA). Naive B cells were isolated by negative selection using an LD column and MidiMACS Separator (Miltenyi Biotec, Bergisch Gladbach, DE) with anti-CD43 (Ly48) mouse microbeads (Miltenyi Biotec, Bergisch Gladbach, DE). B cells were cultured at a density of 5 × 10^6^ cells/mL in full RPMI (RPMI 1640 medium [Sigma-Aldrich, St. Louis, MO] supplemented with 10% heat-inactivated fetal calf serum, 0.2 mM MEM nonessential amino acids, 50 *μ*M 2-mercaptoethanol, and penicillin-streptomycin-glutamine; all from Gibco, Thermo Fisher Scientific, Waltham, MA).

### IC generation

Blood was collected from a C57BL/6 mouse by cardiac puncture and allowed to clot for 30 min at room temperature. The clot was removed by centrifuging at 2000 × *g* for 10 min at 4°C. The liquid component (serum) was divided into 10 *μ*L aliquots on ice and stored at −20°C.

ICs were generated by mixing 10 *μ*L of the mouse serum, as a source of complement, with 0.5 *μ*g Cy3B-labeled goat IgG1 anti-mouse Igκ (Southern Biotech, Birmingham, AL; labeled in-house), 0.375 *μ*g donkey anti-goat IgG (H + L) (Jackson ImmunoResearch, West Grove, PA), and 40 *μ*L of GVB++ buffer (Complement Technology, Tyler, TX) for 30 min at 37°C.

For investigations between B1-8 B cells and SSMs, ICs were generated by mixing 0.5 *μ*g NIP_4_- or NP_4_-labeled Armenian hamster IgG isotype control antibody (BioLegend, San Diego, CA), 0.375 *μ*g goat anti-Hamster (Armenian) IgG antibody DyLight 649 (BioLegend, San Diego, CA), 10 *μ*L mouse serum, and 40 *μ*L GVB++ buffer for 30 min at 37°C. The Armenian hamster IgG isotype control antibody was labeled in-house using either 4-hydroxy-3-iodo-5-nitrophenylacetic active ester (NIP-Osu; LGC Biosearch Technologies, Teddington, UK) or 4-hydroxy-3-nitrophenylacetic acid active ester (NP-Osu; LGC Biosearch Technologies, Teddington, UK).

### Antibodies for imaging and flow cytometry

Antibodies for cell enrichment, flow cytometry, and fluorescence imaging are listed in Table S1. In-house labeling of antibodies was achieved by mixing NHS ester-coupled dyes with the antibody at a 4:1 dye/antibody ratio in sodium bicarbonate buffer (pH 8.2), for 30 min at room temperature and then removing unbound dye molecules using 7 kDa MWCO Zeba desalting columns (Pierce).

### Cell characterization by flow cytometry

Single cells from the whole lymph node and positive selected fraction were split into 10^6^ cells/tube and blocked with anti-mouse CD16/32 in PBS for 20 min at 4°C and surface stained for 30 min at 4°C with labeled primary antibodies or their isotype controls (see Table S1). Propidium iodide was used to exclude dead cells. Flow cytometry was performed on a BD FACS Canto II. Data were analyzed using FlowJo software (BD Biosciences, Franklin Lakes, NJ).

### Imaging and image processing

Wide-field z-stack and time-lapse fluorescence images were acquired using a Nikon TiE TIRF microscope equipped with a 100×, 1.49-NA oil-immersion objective (Nikon Instruments, Amstelveen, NL), a motorized stage with an integrated piezo Z-drive (MS-2000; Applied Scientific Instrumentation, Eugene, OR), and active Z-drift correction (Perfect Focus System; Nikon Instruments, Amstelveen, NL). The microscope was controlled by a high-speed TTL, I/O, DAC controller (Triggerscope 4; Cairn Research, Faversham, UK) integrated into MicroManager Software. Illumination was supplied by a MultiLine LaserBank (Cairn Research, Faversham, UK) fitted with 405-, 488-, 561-, and 640-nm diode lasers (Coherent OBIS). The beams were aligned into a single-mode fiber and coupled to an iLas2 Targeted Laser Illuminator (Gataca Systems, Massy, FR), which produces a 360° spinning beam with an adjustable illumination angle. Laser beams were passed through a laser quadband (405/488/561/640 nm) filter set for TIRF applications (TRF89901v2-ET; Chroma Technology, Bellows Falls, VT) before illuminating the sample. Emitted fluorophores were filtered by appropriate single-band emission filters (Chroma Technolgoy, Bellows Falls, VT) using a filter wheel (OptoSpin; Cairn Research, Faversham, UK) and then captured onto a back-illuminated sCMOS camera (Prime 95B sCMOS; Teledyne Photometrics, Tucson, AZ). For live-cell imaging, a constant temperature of 37°C was maintained using a cage incubator (Okolab, Pozzuoli, IT). Images were processed and analyzed using Fiji (30) and Icy v1.9.9.1 (31). Briefly, images from each channel were aligned and cropped to remove poorly illuminated areas at the edges. Images were then background-subtracted and flat-field corrected before analysis.

Confocal z-stack images were acquired using a Nikon A1R + inverted confocal microscope equipped with a 60×, 1.4-NA oil-immersion objective (Nikon Instruments, Amstelveen, NL). Illumination was supplied by a 488-nm diode laser. Images were reconstructed using NIS Elements software.

### Plots and statistics

Data for individual cells and the mean values per experiment were plotted together as SuperPlots (32). Statistical tests were performed using GraphPad Prism software (version 10), and differences were considered to be statistically significant at p ≤ 0.05.

### Preparation of glass substrates

Stock solutions of collagen were prepared by dissolving 10 mg collagen I (rat tail derived; Roche, Basel, CH) in 3.3 mL of 0.2% (v/v) acetic acid in water. The resulting 3 mg/mL stock solution was stored at 4°C. On the day of an imaging experiment, the collagen was diluted to 30 *μ*g/mL (unless otherwise specified) in PBS, pH 7.4, and incubated on the 10-mm FluoroDish glass surface for 3 h at 37°C. The collagen was aspirated, and the surfaces were incubated with full DMEM for 20 to 30 min at 37°C, before cells were seeded. A similar procedure was used to coat glass coverslips with fibronectin (Roche, Basel, CH) and laminin (Gibco, Thermo Fisher Scientific, Waltham, MA).

### Preparation of polyacrylamide gels

The glass surfaces of 10-mm FluoroDishes were each incubated with 100 *μ*L of 0.1 M sodium hydroxide for 5 min, washed with ultrapure water (Arium), and dried. The surfaces were then incubated with 100 *μ*L of 1.5% (v/v) (3-aminopropyl)trimethoxysilane in ultrapure water for 30 min at room temperature. The surfaces were washed again and incubated with 100 *μ*L of 0.5% glutaraldehyde in PBS for 30 min, washed with ultrapure water, and dried. To generate gels of different stiffness, different concentrations of acrylamide and bis-acrylamide (see Table S3) were mixed with 0.1% ammonium persulfate and 0.1% N,N,N′,N′-tetramethylethylenediamine in degassed 10 mM HEPES (pH 7.0). Two microlitres of the solution were promptly placed in the center of the FluoroDish glass surface and covered with a 9-mm round glass coverslip (made hydrophobic by prior treatment with Rain-X). Two magnets were used to hold the glass-gel-glass sandwich together during polymerization to ensure uniform gel thickness. After 30 min the polymerization was complete, the round coverslips were removed, and the gels were soaked in 50 mM HEPES (pH 7.0). To coat the gel surfaces with collagen, they were first incubated with 100 *μ*L of 0.5 mg/mL sulfo-SANPAH (sulfosuccinimidyl 6-(4′-azido-2′-nitrophenylamino)hexanoate), Thermo Fisher Scientific, Waltham, MA) in 10 mM HEPES (pH 8.5), and exposed to 6 W of 365 nm UV light (UVP UVL-56 handheld UV lamp) until the solution turned from orange to brown. Excess sulfo-SANPAH was removed by washing with 50 mM HEPES (pH 8.5). Crosslinked gels were then incubated with 100 *μ*L of 30 *μ*g/mL collagen I diluted in 50 mM HEPES (pH 8.5), for 2 h at 37°C. Gels were washed with full DMEM and incubated for 20–30 min at 37°C before adding cells.

### Measurements of polyacrylamide gel stiffness

The stiffness (Young’s modulus, *E*) of polyacrylamide gels was measured by nanoindentation using an atomic force microscope (BioScope Resolve; Bruker, Billerica, MA). In brief, cantilevers with pyramidal silicon nitride tips with an effective half-angle θ of 18°, nominal spring constant of k = 0.03 N m^−1^, nominal tip radius of 20 nm, and minimal tip height of 2.5 *μ*m (MLCT-D; Bruker, Billerica, MA) were used. The AFM was calibrated using the deflection sensitivity and actual spring constant of the cantilevers. To determine the deflection sensitivity of the cantilevers, a deflection/force curve for the approach on a glass surface was recorded. The actual spring constant was found through thermal tuning using the simple harmonic oscillator model (33).

Two gels per stiffness were measured. For each gel, measurements were obtained from four areas around the center, spaced at least 4 *μ*m apart. Per area, 64 (8 × 8) force-displacement curves with a ramp size of 5 *μ*m and ramp speed of 10 *μ*m s^−1^ were recorded. The Young’s modulus was then computed using a model for force-indentation relationships of a four-sided pyramidal tip (34). All the force curves were processed in this way with a custom-written MATLAB code.

### Immunofluorescence imaging of fixed cells

For immunofluorescence imaging, cells were exchanged into ice-cold HBSS 0.1% BSA, incubated with ICs for 10 min on ice and fixed with 4% paraformaldehyde. Cells were then blocked with anti-mouse CD16/32 and stained for surface markers using antibodies at a final concentration of 1 *μ*g/mL for 30 min at room temperature. For staining cytoplasmic molecules, cells were permeabilized with 0.1% Triton X-100 (Alfa Aesar, Thermo Fisher Scientific, Waltham, MA) for 5 min in HBSS 0.1% BSA, blocked with 5% (v/v) normal mouse serum (Jackson ImmunoResearch, West Grove, PA), and incubated with the antibody for intracellular staining. F-Actin was stained using Alexa Fluor 488 phalloidin (Invitrogen, Thermo Fisher Scientific, Waltham, MA). Cells were fixed again before imaging.

Because jasplakinolide competitively inhibits phalloidin binding to F-actin, preventing the combined use of these drugs during imaging experiments (35), we used a silicon rhodamine (SiR)-jasplakinolide conjugate. At low concentrations SiR-jasplakinolide can be used as a cell-permeable probe to study actin dynamics, but at the 100 nM concentration we used, it potently induced actin polymerization and inhibited actin turnover while also allowing us to visualize actin filaments.

### Analysis of cell morphology

The cell body was segmented based on the intensity of the F-actin labeling. The cell shape, circularity and volume were determined in Icy using the region of interest (ROI) statistics plugin. The podosome size and filopodia length were measured in Fiji. In brief, F-actin signals corresponding to podosomes were thresholded, binarized, and applied as a mask to the F-actin raw image, and the Analyze Particles plugin was used to quantify podosome number, size, and fluorescence intensity. Filopodia lengths in SSMs cultured on different substrates were measured manually, using the Fiji line selection tool, by a researcher knowledgeable of the actin structures but blinded to the data set (i.e., which substrate cells were adhered to).

### Fluorescence intensity-based colocalization analysis of ICs and cytoskeletal filaments

Our approach to quantify the association of ICs (spots) and F-actin (filaments) was inspired by Sun et al. (36). In brief, two-color z-stack epifluorescence images (phalloidin and IC channels) were processed and analyzed by a user-guided pipeline implemented in Fiji (30). There were three steps to the analysis: (1) identify the z-positions of the dorsal membrane ruffles, (2) remove low-frequency signals from F-actin and IC channels, and (3) quantify the spatial association of ICs and F-actin.

Because dorsal membrane ruffles and ICs in different regions of the cell come into focus at different z-stack positions, six ROIs were analyzed for each cell. The Sobel transform and Canny-Deriche operator were applied to the phalloidin channel, and the three adjacent z-slices with the highest integrated values (sharpest frames), corresponding to the z-slices with dorsal membrane ruffles, were identified. A maximum intensity projection of the three z-slices from the phalloidin raw image stack was generated and used for further analysis. A maximum intensity projection of the same three z-slices was produced from the IC raw image stack.

Next, segmented images of dorsal membrane ruffles and ICs were generated. For each channel (phalloidin and ICs), low-frequency signals were subtracted (using a Gaussian bandpass filter in the phalloidin channel and a Laplacian of Gaussians filter in the IC channel) and then each channel was thresholded, binarized, and subsequently applied as a mask to the raw phalloidin and IC channels to exclude background fluorescence signal. The ICs were identified, and the raw integrated density of each complex quantified, using the Analyze Particles plugin. The enrichment ratio of ICs on actin filaments, R, was then calculated for each ROI as the summed intensity of ICs that are "on actin," I_c,on_, divided by the summed intensity of all ICs, I_c,on+off_, or

$$R=\frac{\sum I_{c,\text{on}}}{\sum I_{c,\text{on}+\text{off}}}$$

The same procedure was used to calculate the enrichment ratio for ICs with microtubules, vimentin, and nestin. For these experiments, antibodies targeting the cytoskeletal proteins were used in place of phalloidin.

### Analysis of B cell antigen internalization from SSMs

For quantification of antigen uptake from SSMs, B cells were incubated on SSMs cultured on collagen I-coated substrates for 20 min at 37°C. Image z-stacks of fixed cells were processed and analyzed as described previously (11). In brief, antigen internalization was quantified by detecting antigen clusters extracted from SSMs inside the B cells, using B220 surface staining to segment the B cells. Images were bandpass filtered and antigen clusters were identified using a user-specified global intensity threshold.

### Single-particle tracking and analysis

Cells cultured on collagen I-coated substrates were incubated with fluorescent ICs for 10 min on ice, exchanged into warm HBSS 0.1% BSA, and imaged at 37°C. Streamed time-lapse images were acquired at a frame rate of 20 Hz for 15 s at a single plane focused on the dorsal cell membrane. Images were background-subtracted and corrected for photobleaching using either the histogram matching method (IC channel) or the exponential fitting method (LifeAct-green fluorescent protein [GFP] channel) in Fiji (37). A Difference of Gaussians filter was applied to the IC image (σ_1_: 3.00 and σ_2_: 1.50). The particles were detected and tracked with subpixel localization using the LAP tracker in the Trackmate plugin for Fiji (38), with a maximum linking distance of 500 nm, a maximum gap-closing distance of 250 nm, and a maximum gap of 2 frames.

For the kymograph analysis, single-particle signals were extracted along the IC trajectory. Kymographs were then generated from videos of 300 frames using the Multi Kymograph plugin for Fiji, with a line width of 1 pixel. The evolution of fluorescence intensity along the IC trajectories was plotted versus time to generate the kymographs for both the IC and F-actin channels.

The MSD of each IC diffusing in two dimensions, in the plane of the plasma membrane, was computed according to the formula (39).

$$\text{MSD}\left(\tau \right)=\left\langle {\left[r\left(t+\tau \right)-r\left(t\right)\right]}^{2}\right\rangle =4D\tau^{\alpha }$$

where τ=50ms is the frame time, [r(t+τ)−r(t)] is the IC displacement during time interval *τ*, *D* is the apparent diffusion constant, and *α* is the anomalous scaling exponent, with α=1 characterized as Brownian (normal) diffusion, α<1 as subdiffusion, and α>1 as superdiffusion. Trajectories of 10 spots or longer were used for the analysis. Linear trajectories due to active transport of internalized ICs were excluded from the analysis.

To identify different mobility states sampled by individual ICs, their trajectories were analyzed using SMAUG as described previously (40). The chord diagrams in Fig. 4 *F* were generated using Fluorish (https://flourish.studio).

### Live-cell imaging of SSM-B cell interactions

SSMs cultured on collagen I-coated glass coverslips were treated with 3 *μ*M mycalolide B (ChemCruz) or DMSO in HBSS 0.1% BSA for 30 min at 37°C. SSMs were then incubated with fluorescent ICs on ice for 10 min and washed three times with warm HBSS 0.1% BSA to remove mycalolide B from the wells. Naive B cells were preloaded with 5 *μ*M Cal-520-AM for 2 h at 37°C, washed with warm HBSS 0.1% BSA, and added at a density of 0.5 × 10^6^ cells per well to interact with SSMs. Time-lapse images were acquired at 37°C with 50 ms exposure time and 20 s between frames for 80 frames. At each time point, images were acquired sequentially in the 488- and 561-nm channels to visualize Cal-520 and Cy3B-ICs, respectively. Images were acquired at a single z-plane focused on the dorsal side of the SSM to capture interactions with B cells. B cells were tracked in the Cal-520 channel using the LAP tracker in the Trackmate plugin for Fiji, with a maximum linking distance of 7 *μ*m, a maximum gap-closing distance of 3.5 *μ*m, and a maximum gap of 2 frames.

### NF-κB nuclear translocation assay

SSMs cultured on collagen I-coated substrates were incubated with fluorescent ICs on ice for 10 min and then washed with warm HBSS 0.1% BSA. B cells were then added at a density of 1.5 × 10^6^ cells per well and allowed to interact with SSMs for 20 min at 37°C before fixation with 2% PFA. Wells were blocked with normal mouse serum (Jackson ImmunoResearch, West Grove, PA) before surface staining with B220 BV421, permeabilization, and intracellular staining with anti-NF-κB1 p65. Cells were then incubated with the secondary antibody anti-rabbit IgG (H + L) F(abʹ)_2_ Alexa Fluor 647 and with DAPI to stain nuclei. Cells were fixed again and imaged. Nuclear translocation of p65 for each cell was assessed by three knowledgeable researchers who were blinded to the identification of the data sets.

### Code availability

The ImageJ/FIJI macros used to perform the Enrichment Analysis are available on GitHub: https://github.com/maroiliop/Enrichment-Analysis/blob/main/README.md.

## Results

### Identifying SSMs in vitro

We identified SSMs in culture experiments fortuitously while attempting to isolate FDCs from mouse lymph nodes. FDCs are stromal cells that we and others have previously isolated by positive selection using antibody complexes containing an FDC-specific antibody (rat IgG2c, κ anti-mouse FDC-M1) (15,41,42). This approach enriches FDCs but does not purify them, and FDCs must be identified in culture as CD45^−^ CD21/35^+^ cells that capture and retain ICs on their surfaces. During our investigations, we identified a population of CD45^+^ CD21/35^−^ cells that were also heavily labeled by ICs. Using a combination of flow cytometry (Fig. S1) and multicolor fluorescence imaging (Fig. S3), we determined—based on an extended CD11b^+^ CD11c^−^ CD68^+^ CD169^+^ F4/80^neg/low^ CD209b^−^ phenotype and ability to display large amounts of ICs—that the cells were SSMs (26,43,44,45).

### SSMs use Fcγ receptors to present ICs

SSMs do not express FDC-M1 but do express high levels of Fcγ receptors (FcγRs) (46). We therefore postulated that SSMs were enriched during the cell isolation procedure due to interactions between their FcγRs and the Fc domain of the rat IgG2c, κ anti-mouse FDC-M1 primary antibody, or the biotinylated mouse IgG2a anti-rat Igκ secondary antibody used for cell enrichment. First, we confirmed that cells expressed FcγRs by staining with anti-CD16/32 (FcγRIII/FcγRII) (Fig. S4 *A*). Next, we repeated the cell isolation using a rat IgG2c, κ isotype control antibody in place of the FDC-M1 antibody. Flow cytometric analysis confirmed our hypothesis that SSMs were enriched through interactions between IgG antibody complexes and FcγRs (Fig. S1 *C*). We propose that using IgG antibody complexes as bait to enrich SSMs from heterogeneous lymph node populations is a gentle and efficient method to attain these cells for functional in vitro studies.

Given the high capacity of SSMs to bind the Fc domain of IgG antibodies, we wondered whether these cells require complement to present ICs. To answer this question, we loaded SSMs with antibody complexes composed of goat IgG anti-mouse κ and donkey anti-goat IgG (H + L) that either were, or were not, mixed with serum in GVB++ buffer as a source of complement. We observed that complement fixation was not required for SSMs to capture and retain antibody complexes on the cell surface (Fig. S4, *B* and *C*). Therefore, we conclude that SSMs primarily use FcγRs to present ICs.

### ICs on the SSM surface colocalize with actin filaments

**Figure 2:**
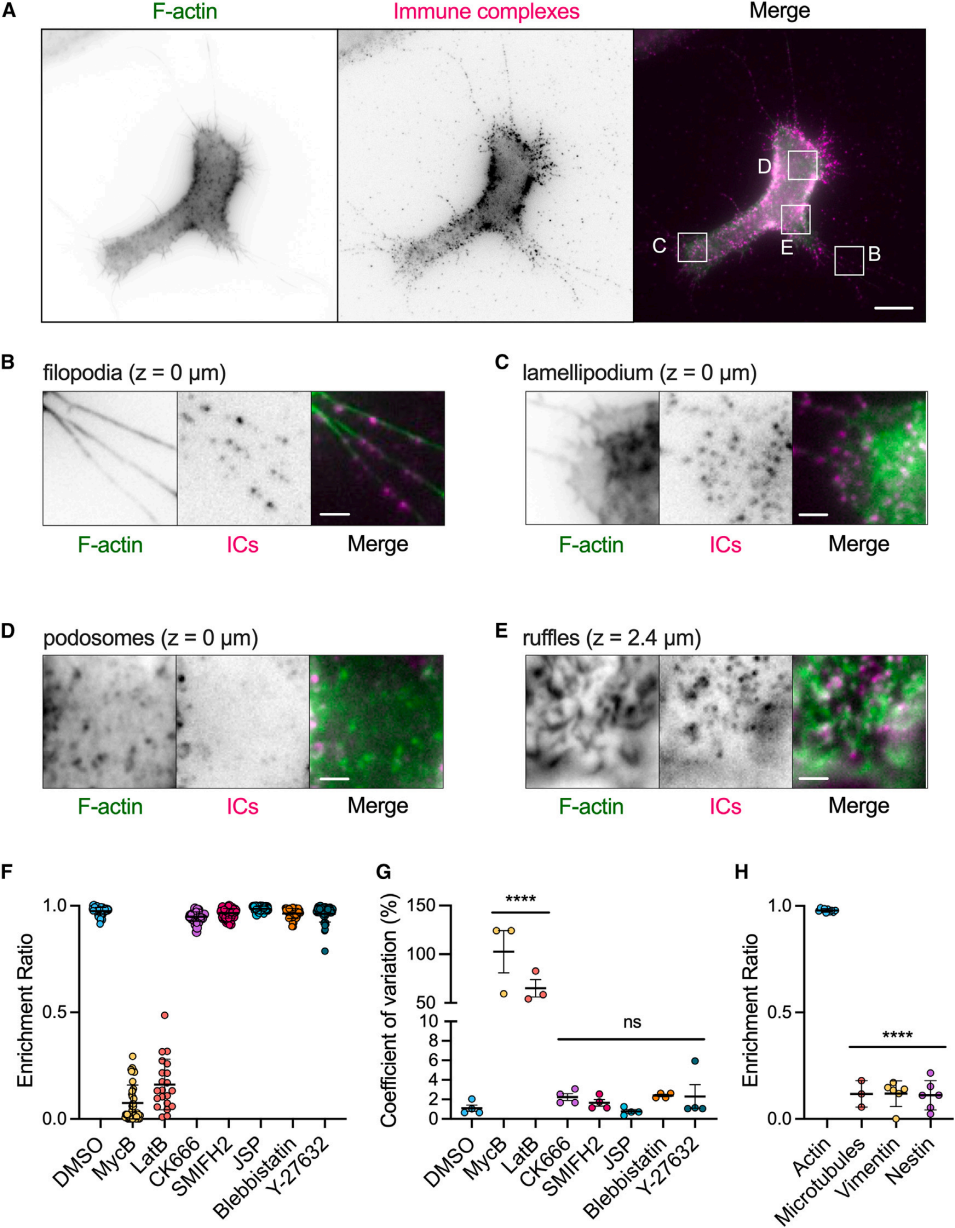
ICs presented by SSMs associate with actin filaments. (*A*) A single z-plane image (z = 0 *μ*m) from a z-stack of an SSM labeled with ICs and stained with phalloidin-AF488. Scale bar, 10 *μ*m. (*B–E*) Zoomed in regions highlighted in (*A*) showing the different F-actin structures formed by SSMs including (*B*) filopodia, (*C*) lamellipodia, (*D*) podosomes, and (*E*) dorsal membrane ruffles. ICs densely label filopodia, lamellipodia, and ruffles and are excluded from podosomes. Scale bars, 2 *μ*m. (*F*) Quantitation of the spatial association of ICs with dorsal membrane ruffles in SSMs treated with drugs that perturb actin filament assembly or actomyosin contractility. An enrichment ratio value of 1 indicates "on actin" and a value of 0 indicates "off actin.” Each dot represents the mean enrichment ratio value of all ICs within six regions of interest (ROIs) from one cell. *n* = 21–52 cells per treatment, pooled from five independent experiments. Bars represent mean ± SD. (*G*) Coefficient of variation values for the data in (*F*). Each dot represents the mean value from one experiment. Bars represent mean ± SEM. (*H*) Quantitation showing that ICs associate with F-actin-rich dorsal membrane ruffles, but not microtubules, vimentin, or nestin. Bars represent mean ± SD. ns, not significant. ^∗∗∗∗^*p* < 0.0001, comparing drug-treated cells to DMSO-treated cells (*G*) or IC association with microtubules and intermediate filaments compared with F-actin (*H*) (one-way ANOVA with Dunnett’s multiple comparisons test). To see this figure in color, go online.

We next characterized the morphology of SSMs at the single-cell level in vitro. To do this, we cultured SSMs on collagen I-coated glass coverslips, loaded them with Cy3B-labeled ICs, and then fixed, permeabilized, and stained them with phalloidin-Alexa Fluor 488 (AF488) to detect filamentous (F)-actin (Fig. 2 *A*). The phalloidin staining revealed that SSMs formed several distinct F-actin-based structures including filopodia (Fig. 2 *B*), lamellipodia (Fig. 2 *C*), and podosomes (Figs. 2 *D* and S5) at the cell-ECM interface and actin-enriched membrane ruffles (Fig. 2 *E*) on the dorsal cell surface. ICs were excluded from the actin core of podosomes but colocalized with both filopodia and ruffles, and heavily decorated lamellipodia.

We quantified the spatial association of ICs with F-actin, focusing on membrane ruffles. To do this, we developed an image analysis workflow that assigned each IC on the dorsal cell surface as either “on actin” or “off actin” to determine the fraction of ICs associated with dorsal actin filaments (see materials and methods). The quantitation showed that all ICs on the dorsal cell surface were associated with membrane ruffles (Fig. 2, *E* and *F*; Video S1). Treatment of the cells with pharmacological inhibitors of proteins that regulate actin assembly or contractility, including jasplakinolide (actin nucleation), blebbistatin (myosin II), and Y-27632 (Rho-associated protein kinase), did not influence IC-actin association (Figs. 2, *F*, *G*, and S6 *A*; Videos S2, S3, and S4) and had differential effects on cell size and shape (Fig. S6, *B–D*). SMIFH2 (formins) and CK-666 (Arp2/3) reduced the fraction of SSMs with membrane ruffles (Fig. S6 *E*) and the total F-actin content in the cells (Fig. S6 *F*). Yet, the cells retained other F-actin structures on their dorsal surfaces that colocalized with ICs, and we considered these complexes to be “on actin” in our analysis. Specifically, CK-666-treated cells contained actin filaments that protruded from the cell surface (Video S5) and SMIFH2-treated cells showed more branched actin structures at the dorsal membrane (Video S6). These changes reflect the known competition between Arp2/3 and formins for actin monomers (47). Only mycalolide B and latrunculin B, drugs that sever actin filaments, completely disrupted the actin-IC association. The loss of F-actin led to the formation of large IC aggregates (Fig. S6 *A*), suggesting that F-actin regulates the spatial organization of ICs on the cell surface. We also confirmed that the association of ICs with cytoskeletal filaments was specific to F-actin; ICs did not colocalize with microtubules or the intermediate filaments vimentin and nestin (Figs. 2 *H* and S7, *A–C*). These results demonstrate that ICs have a strong propensity to localize to F-actin-based membrane protrusions in SSMs.

Video S1. Raw epifluorescence z-stack of a DMSO-treated SSM cultured on collagen I-coated glass, labeled with phalloidin-AF488 (*green*) and Cy3B-ICs (*magenta*) Image size: 76.45 × 76.45 *μ*m (695 × 695 pixels). Z-step size: 0.5 *μ*m.

Video S2. Raw epifluorescence z-stack of a jasplakinolide-treated SSM cultured on collagen I-coated glass, labeled with phalloidin-AF488 (*green*) and Cy3B-ICs (*magenta*) Image size: 76.45 × 76.45 *μ*m (695 × 695 pixels). Z-step size: 0.5 *μ*m.

Video S3. Raw epifluorescence z-stack of a blebbistatin-treated SSM cultured on collagen I-coated glass, labeled with phalloidin-AF488 (*green*) and Cy3B-ICs (*magenta*) Image size: 76.45 × 76.45 *μ*m (695 × 695 pixels). Z-step size: 0.5 *μ*m.

Video S4. Raw epifluorescence z-stack of a Y-27632-treated SSM cultured on collagen I-coated glass, labeled with phalloidin-AF488 (*green*) and Cy3B-ICs (*magenta*) Image size: 76.45 × 76.45 *μ*m (695 × 695 pixels). Z-step size: 0.5 *μ*m.

Video S5. Raw epifluorescence z-stack of a CK-666-treated SSM cultured on collagen I-coated glass, labeled with phalloidin-AF488 (*green*) and Cy3B-ICs (*magenta*) Image size: 76.45 × 76.45 *μ*m (695 × 695 pixels). Z-step size: 0.5 *μ*m.

Video S6. Raw epifluorescence z-stack of a SMIFH2-treated SSM cultured on collagen I-coated glass, labeled with phalloidin-AF488 (*green*) and Cy3B-ICs (*magenta*) Image size: 76.45 × 76.45 *μ*m (695 × 695 pixels). Z-step size: 0.5 *μ*m.

### IC mobility coincides with F-actin dynamics

**Figure 3:**
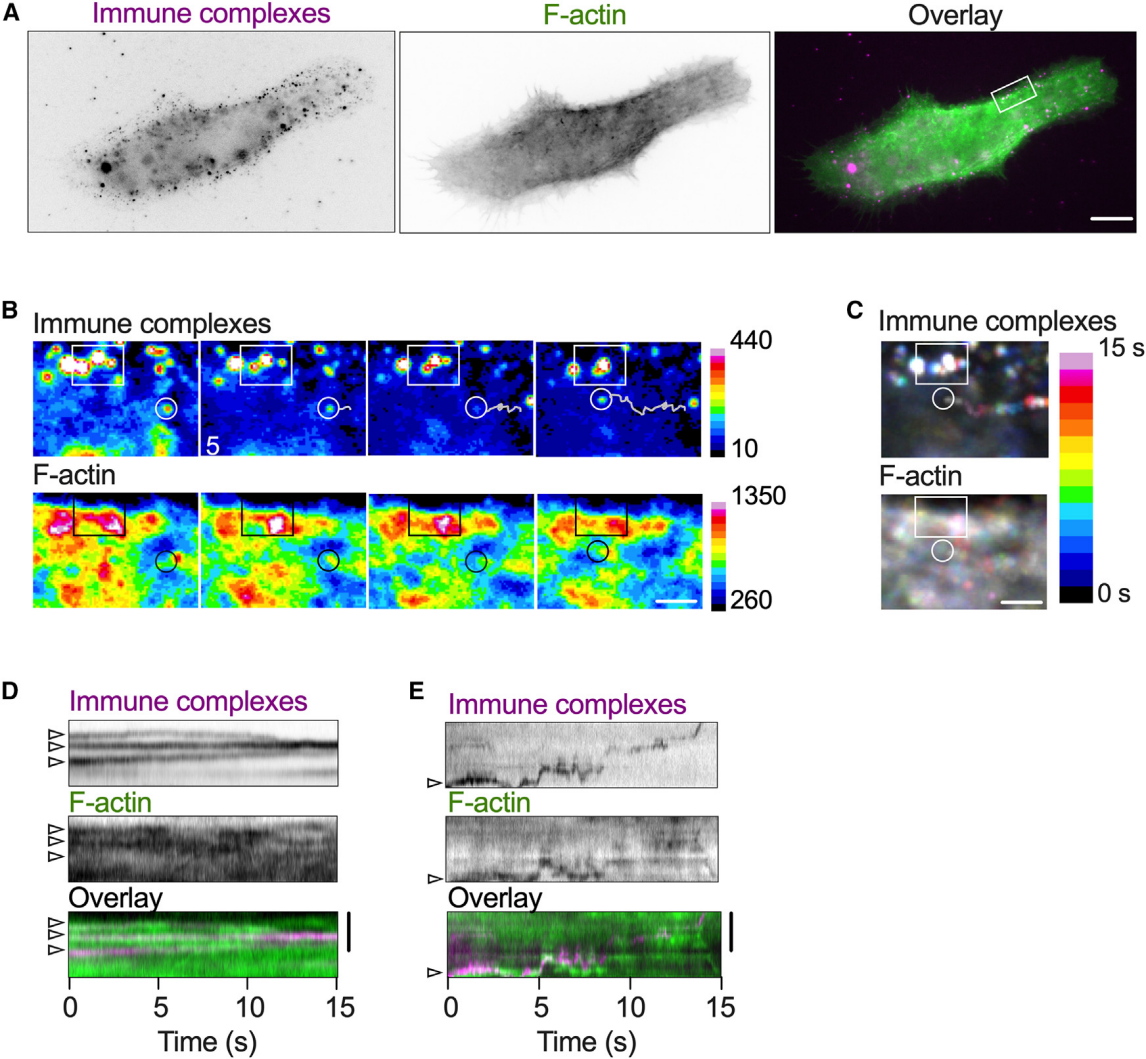
The movement of F-actin and ICs are spatiotemporally correlated at the SSM surface. (*A*) Representative wide-field image of F-actin (LifeAct-GFP, *green*) and ICs (Cy3B-labeled, *magenta*) at the dorsal membrane of a live SSM. Scale bar, 10 *μ*m. (*B*) Magnification of the boxed area in (*A*). Two-color time-lapse images of ICs and F-actin. Images are pseudo colored to represent changes in fluorescence intensity. Low-mobility ICs are denoted by a white box and by the corresponding black box in the F-actin images. These complexes were trapped in a region of high F-actin intensity. A high-mobility IC is denoted by a white circle and the corresponding black circle in the F-actin images. The trajectory traces the movement of the mobile IC across time. The high F-actin intensity immediately adjacent to the complex indicates that the complex is "pushed" by a dynamic F-actin structure. Scale bar, 2 *μ*m. (*C*) Temporal projections of IC and F-actin dynamics (t = 0 to 15 s) with cold colors representing early times and warm colors later times. Scale bar, 2 *μ*m. (*D* and *E*) Kymographs representing the motion of ICs and F-actin from (*D*) the boxed region and (*E*) along the trajectory marked in (*B*). Arrow heads show the starting positions of ICs in each kymograph. Scale bars, 2 *μ*m (vertical). To see this figure in color, go online.

The actin cytoskeleton regulates the functions of immunoreceptors in many immune cell types including phagocytic macrophages, where binding of IgG antibodies to FcγRs induces cytoskeletal remodeling and consequent changes in FcγR mobility and clustering (48). Since we observed that F-actin tightly associated with FcγR-presented ICs in fixed SSMs, we reasoned that the cell cytoskeleton might also constrain the lateral mobility and clustering of ICs in live SSMs. To investigate this possibility, we analyzed the motion of fluorescent ICs on the dorsal surfaces of SSMs expressing LifeAct-GFP (Fig. 3 *A*), which binds specifically to F-actin in live cells without interfering with actin dynamics (49). IC labeling was performed at low density so that individual ICs could be detected. The cell surface was imaged in the IC and F-actin channels with a frame rate of 20 Hz for 15 s using wide-field epifluorescence microscopy at 37°C (Video S7).

Video S7. Raw epifluorescence time-lapse of Cy3B-labeled ICs diffusing in the plasma membrane of a mouse SSM expressing Lifeact-GFP The still images in Fig. 3, *B* and *C*, are taken from this movie. Image size: 252 × 69 pixels, pixel size: 110 nm.

In agreement with the fixed-cell images, the live-cell time-lapse images showed that ICs accumulated near regions of high F-actin intensity (Fig. 3 *B*). The motion of ICs and F-actin structures was correlated; slow-moving ICs associated with large, static F-actin aggregates while highly mobile ICs appeared to be “pushed" by small, dynamic F-actin patches (Fig. 3, *B* and *C*; Video S7). The coincidence of IC and F-actin dynamics was further supported by kymographic analysis (Fig. S8). Within dense regions of F-actin, low-mobility ICs were brought together to form larger clusters, indicating that F-actin could induce clustering of ICs on the cell surface (Fig. 3 *D*). Elsewhere on the cell membrane, fast-moving ICs and F-actin patches showed that they were spatiotemporally linked (Fig. 3 *E*). Taken together, these observations indicate that the SSM actin cytoskeleton regulates the organization and mobility of ICs on the cell surface.

### ICs presented by SSMs exhibit multiple diffusive states

**Figure 4:**
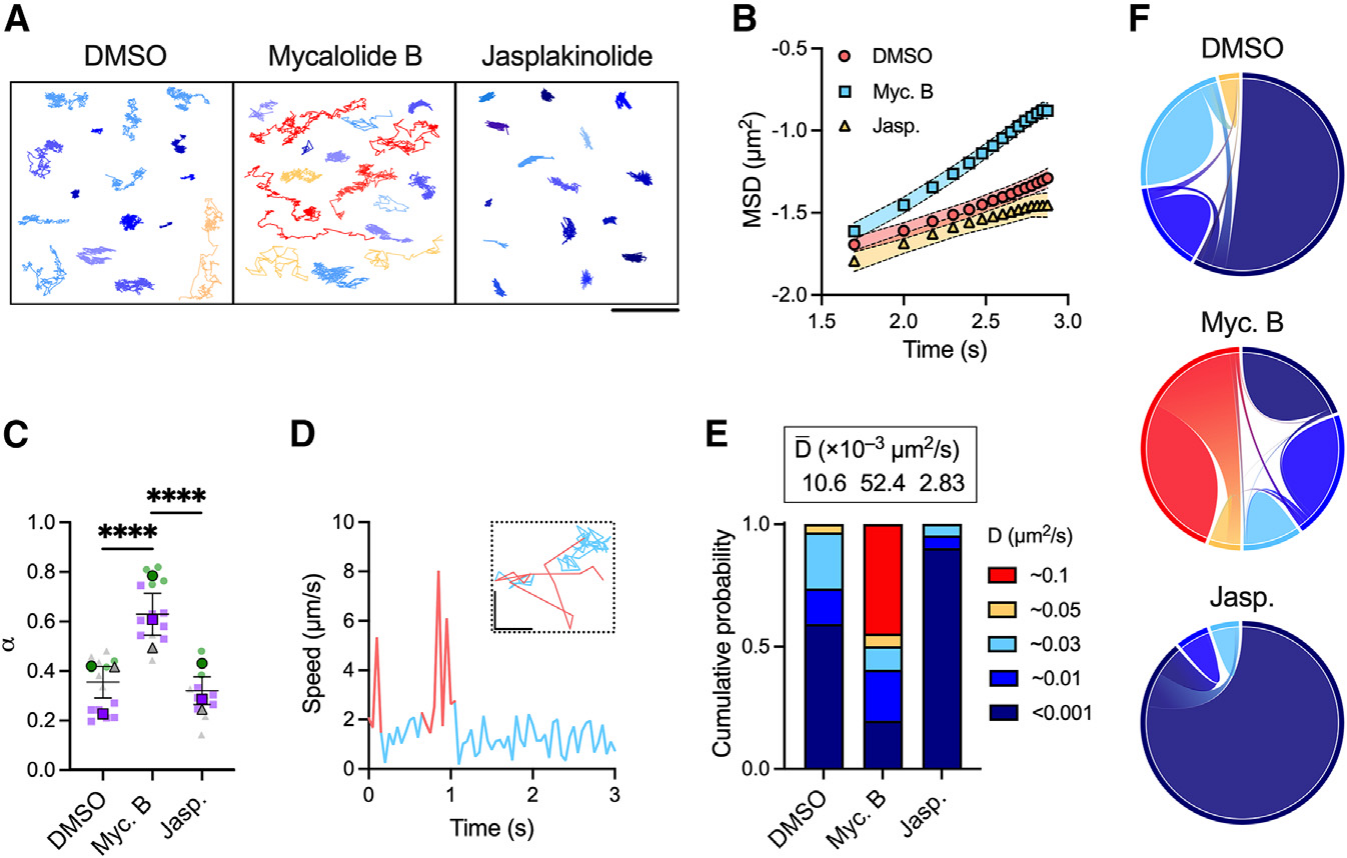
Quantitative analysis of IC single-particle trajectories. (*A*) Representative trajectories of individual ICs diffusing on the surfaces of SSMs treated with DMSO, mycalolide B, or jasplakinolide. Trajectories are color coded by their diffusion constant, with highly mobile complexes in red ($D\approx 0.1\;\mu m^{2}/s$) and immobile complexes in dark blue ($D<0.001\;\mu m^{2}/s$). Scale bar, 200 nm. (*B*) Log-log plot of MSD versus time from IC single-particle trajectories. The symbols represent the mean MSD values from all acquired trajectories per condition (>1000 trajectories per cell; DMSO: 13 cells, Myc B: 14 cells, Jasp.: 10 cells), and the fills the standard errors of the mean. Data are from one experiment representative of three experiments. (*C*) Anomalous scaling exponents, *α*, extracted as the slopes from linear fits to plots of MSD versus *τ* for the first 10 time lags. Each plain dot represents the mean *α* value for >1000 ICs from one cell (*n* = 2–7 cells per condition) and each outlined dot represents the mean value for one independent experiment (*N* = 3 experiments). Data are color coded by experiment. Bars represent mean ± SEM. (*D*) Sample plot of instantaneous speed versus time for one IC. Instances of high speed are color-coded pink and instances of low speed are blue. The corresponding *xy* trajectory is shown in the inset with the same color-coding scheme. Scale bars, 200 nm (inset). (*E*) Bar graphs showing the mean weight fraction, *π*, of each mobility state identified by SMAUG analysis for ICs diffusing on cells treated with DMSO, mycalolide B, and jasplakinolide. The plot was constructed from the same single-particle trajectories used to calculate the values of *α* in (*C*) and merges data from three independent experiments. The weighted mean of the diffusion constant for each condition, $\bar{D}$, is given atop each bar. ^∗∗∗∗^*p* < 0.0001 (one-way ANOVA with Tukey’s multiple comparisons test). (*F*) Chord diagrams depicting the transition probabilities between mobility states for cells treated with DMSO, mycalolide B, and jasplakinolide. Each band represents a transition between two mobility states, and the width of a band indicates the probability that the transition will occur. The colors encode the diffusion constants for the different states as shown in (*E*). To see this figure in color, go online.

The previous results showed that ICs spatially and temporally associated with F-actin in SSMs expressing LifeAct-GFP. To explore further the role that F-actin plays in controlling IC motion, we characterized the diffusion of ICs displayed by cells that had been treated with mycalolide B to depolymerize actin filaments, jasplakinolide to stabilize actin filaments, or DMSO as a control. Visual inspection of the IC trajectories with and without drug treatments indicated that the SSM actin cytoskeleton did influence IC motion (Fig. 4 *A*). In control (DMSO-treated) cells, we observed immobile and partly mobile populations as before (Fig. 3, *A–E*). Treatment with mycalolide B visibly increased IC mobility (Fig. 4 *A*; Video S8) and treatment with jasplakinolide immobilized all ICs (Fig. 4 *A*; Video S9).

Video S8. Raw epifluorescence time-lapse of Cy3B-labeled ICs diffusing in the plasma membrane of a mouse SSM treated with mycalolide B Image size: 90 × 90 pixels, pixel size: 110 nm.

Video S9. Raw epifluorescence time-lapse of Cy3B-labeled ICs diffusing in the plasma membrane of a mouse SSM treated with fluorogenic jasplakinolide (SiR-Actin) Image size: 180 × 90 pixels, pixel size: 110 nm.

The diffusion of a molecule can be categorized as one of three types based upon the relationship between its mean-squared displacement (MSD), $\left\langle r^{2}\right\rangle$ , and time lag, *τ*: Brownian (random), superdiffusive (directed), and subdiffusive (confined). The MSD of particles diffusing in the plane of a membrane is described by the power law $\left\langle r^{2}\left(\tau \right)\right\rangle =4D\tau^{\alpha }$, where *D* is the diffusion constant and *α* is the anomalous scaling exponent. Values of α=1 indicate Brownian motion, α<1 subdiffusive motion, and α>1 superdiffusive motion. A log-log plot of MSD versus *τ* yields a straight line of slope *α* and thus provides a convenient representation of the nature of the motion (Fig. 4 *B*). Our results showed that IC diffusion was confined in control cells (α=0.38) (Fig. 4 *C*), in agreement with observations that FcγRs are confined within submicron actin compartments in bone marrow-derived macrophages (48). Treatment of SSMs with jasplakinolide to stabilize actin filaments slightly increased IC confinement (α=0.35) while treatment with mycalolide B to remove actin barriers drastically reduced confinement (α=0.62). Thus, our data indicate that the actin cytoskeleton controls the lateral mobility of ICs on the SSM surface.

MSD analysis provides a single diffusion constant for each IC. Our observations that ICs could transiently associate with actin, however, suggested that each complex could sample different mobility states within a single trajectory while remaining, on average, globally confined (Figs. 3 *E* and 4 *A*). This observation was supported by plotting the instantaneous velocity of ICs over time, which revealed that they generally moved with slow speeds of <2 *μ*m/s but were transiently free to move with speeds of 5–8 *μ*m/s (Fig. 4 *D*). To quantify this heterogeneous behavior and better understand the role of actin in generating distinct mobility characteristics, we analyzed the single-particle trajectories using single-molecule analysis by unsupervised Gibbs sampling (SMAUG) (40). SMAUG uses nonparametric Bayesian statistics to uncover different mobility states from within single trajectories and quantify their diffusion constants (*D*) and weight fractions (*π*). We applied this analysis tool to our trajectories and found that, in DMSO-treated cells, most ICs were immobile ($\pi_{\text{immobile}}=0.6$ and $D_{\text{immobile}}<0.001\;\mu m^{2}/s$), while the remaining had moderate mobility ($\pi_{\text{moderate}}=0.4$, $D_{\text{moderate}}=0.01-0.05\;\mu m^{2}/s$) (Fig. 4 *E*). Treatment with mycalolide B to sever actin filaments allowed ICs to diffuse more freely. While the fraction of ICs with moderate mobility was slightly lower ($\pi_{\text{moderate}}=0.35$), the proportion of immobile ICs was substantially reduced ($\pi_{\text{immobile}}=0.2$) and a new, highly mobile population appeared ($\pi_{\text{high}}=0.45$, $D_{\text{high}}=0.1\;\mu m^{2}/s$). Treatment with jasplakinolide to stabilize actin filaments caused almost complete immobilization of ICs ($\pi_{\text{immobile}}=0.9$). For all conditions, there were negligible transitions between the fastest and slowest mobility states (Fig. 4 *F*). Instead, ICs were most likely to transition from a fast state to an intermediate state to a slow state, or vice versa. Taken together, these data indicate that the SSM actin cytoskeleton constrains the mobility of ICs presented by FcγRs on the cell surface.

### ECM composition affects SSM morphology and IC mobility

SSMs are surrounded by a heterogeneous web of ECM proteins that determine the chemical and physical properties of the tissue microenvironment (50,51). To gain a glimpse into how SSMs may respond to different ECM compositions, we measured dose-dependent changes to SSMs adhered to three ECM proteins: collagen I, fibronectin, and laminin. First, we varied the concentration of collagen I over the range 10–30 *μ*g/mL, finding that higher collagen I concentrations led to increased cell area (Fig. S9 *A*) and volume (Fig. S9 *B*) without altering cell shape (Fig. S9 *C*). These changes were accompanied by a dose-dependent increase in F-actin intensity at the dorsal membrane (Fig. S9 *D*), enhanced formation of prominent dorsal membrane ruffles (Fig. S9 *E*), and increased mobility of ICs on the cell surface (Fig. S9 *F*).

We next imaged the cells cultured on fibronectin, laminin, and a mixed matrix comprising 1:2:0.5 collagen I/fibronectin/laminin, which approximates the relative abundance of these ligands in lymph node ECM (52). We tested two concentrations of fibronectin: 10 and 20 *μ*g/mL. We also attempted 10 and 20 *μ*g/mL of laminin, but SSMs did not adhere at the higher laminin concentration. SSM spread area and volume were similar for all ECM ligands at 10 *μ*g/mL concentration (Fig. S9, *A* and *B*), although the mobility of ICs differed ($D_{\text{collagen}\;I}=0.0017\;\mu m^{2}/s$, $D_{\text{fibronectin}}=0.0056\;\mu m^{2}/s$, $D_{\text{laminin}}=0.0085\;\mu m^{2}/s$) (Fig. S9 *F*). Increasing the fibronectin concentration from 10 to 20 *μ*g/mL led to a 90% increase in spread area (Fig. S9 *A*) and 102% increase in cell volume (Fig. S9 *B*). The increased fibronectin content also increased the prominence of dorsal membrane ruffles (Fig. S9, *D* and *E*), although it had no impact on IC mobility—the weighted mean diffusion constant was 0.0056 *μ*m^2^/s at both 10 and 20 *μ*g/mL fibronectin. From these data we conclude that SSMs bind and respond to different ECM ligands in a dose-dependent manner, suggesting that they can adhere to the matrix using different integrin types (53).

### ECM rigidity alters SSM morphology

Our results suggested that dorsal membrane ruffles are morphological structures involved in IC presentation by SSMs. Generally, the morphology of a cell results from the balance of cell-intrinsic forces such as tension and contractility, and cell-extrinsic forces such as ECM stiffness (54). Disrupting the cellular force balance can cause rapid alterations in cytoskeletal structure (55) that can impact cell polarization (56) and membrane ruffling (57). We were therefore curious to know whether SSMs could also alter the organization of their actin cytoskeleton to reflect changes in ECM rigidity.

**Figure 5:**
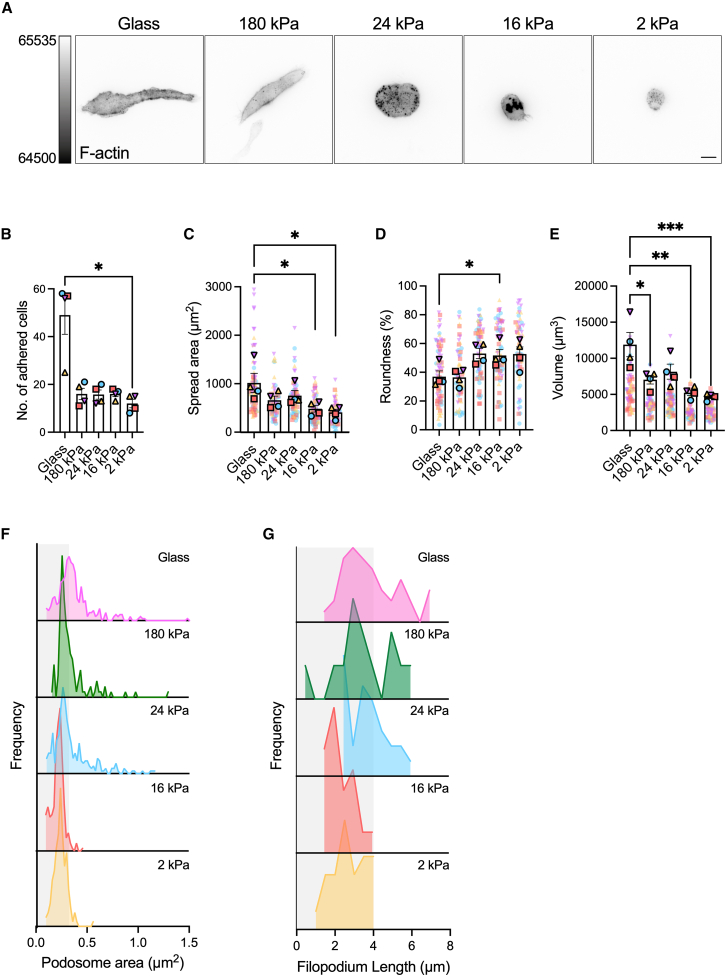
ECM rigidity alters SSM morphology and actin cytoskeleton organization. (*A*) Images of SSMs on collagen I-coated substrates of different stiffness. Scale bar, 10 *μ*m. (*B*) The number of SSMs adhered to the 78.5 mm^2^ substrate surface on day 5 of culture. Data are from four independent experiments. (*C* and *D*) Cells change their size and shape in response to ECM rigidity as assessed by (*C*) spread area (*μ*m^2^), (*D*) roundness (%), and (*E*) volume (*μ*m^3^). Each plain dot represents one cell and each outlined dot represents the mean value for one independent experiment (*n* = 11 to 26 cells per condition per experiment, *N* = 4 independent experiments). Data are color coded by experiment. Bars represent mean ± SEM. (*F* and *G*) Cells respond to changes in substrate stiffness by altering (*F*) the size of podosomes at the ECM-cell interface and (*G*) the length of filopodia. The bin sizes for the histograms in (*F*) and (*G*) are 0.02 *μ*m^2^ and 0.5 *μ*m, respectively. Data are pooled from three or four independent experiments (*n* = 15 to 34 cells per condition per experiment). ^∗^*p* < 0.05, ^∗∗^*p* < 0.01, ^∗∗∗^*p* < 0.001 (one-way ANOVA with Tukey’s multiple comparisons test). To see this figure in color, go online.

To investigate the effect of ECM stiffness on SSMs, we cultured the cells on glass and flat polyacrylamide hydrogels with similar surface densities of collagen I (Fig. S10 *A*) but different rigidities, with mean Young’s moduli ranging from 2 to 180 kPa (Fig. 5 *A*; Table S3; Videos S10, S11, S12, S13, and S14). After 5 days in culture, SSMs were adhered well to all of the gels (Fig. 5 *B*). Imaging F-actin revealed that, compared with cells cultured on glass, cells cultured on 2 kPa gels had a 60% lower mean spread area (Fig. 5 *C*), 43% increased mean roundness (Fig. 5 *D*), and 61% lower cell volume (Fig. 5 *E*). These results indicate that SSMs become smaller and more rounded in response to a softer ECM.

Video S10. Raw epifluorescence z-stack of an SSM cultured on collagen I-coated glass, labeled with phalloidin-AF488 (*green*) and Cy3B-ICs (*magenta*) Image size: 132 × 132 *μ*m (1200 × 1200 pixels). Z-step size: 0.5 *μ*m.

Video S11. Raw epifluorescence z-stack of an SSM cultured on a collagen I-coated 180 kPa gel, labeled with phalloidin-AF488 (*green*) and Cy3B-ICs (*magenta*) Image size: 132 × 132 *μ*m (1200 × 1200 pixels). Z-step size: 0.5 *μ*m.

Video S12. Raw epifluorescence z-stack of an SSM cultured on a collagen I-coated 24 kPa gel, labeled with phalloidin-AF488 (*green*) and Cy3B-ICs (*magenta*) Image size: 132 × 132 *μ*m (1200 × 1200 pixels). Z-step size: 0.5 *μ*m.

Video S13. Raw epifluorescence z-stack of an SSM cultured on a collagen I-coated 16 kPa gel, labeled with phalloidin-AF488 (*green*) and Cy3B-ICs (*magenta*) Image size: 132 × 132 *μ*m (1200 × 1200 pixels). Z-step size: 0.5 *μ*m.

Video S14. Raw epifluorescence z-stack of an SSM cultured on a collagen I-coated 2 kPa gel, labeled with phalloidin-AF488 (*green*) and Cy3B-ICs (*magenta*) Image size: 132 × 132 *μ*m (1200 × 1200 pixels). Z-step size: 0.5 *μ*m.

SSM responses to ECM rigidity were also reflected by changes in the prominence of F-actin-based structures. Podosomes are actin-rich, circular structures that form at the ventral surface of dendritic cells and macrophages, enabling cells to adhere to the ECM via integrins (58). They also have a mechanosensory function, allowing cells to sense the rigidity of the substrate. SSMs on all substrates formed podosomes. Reducing ECM stiffness did not cause a major change in the mean area of the podosome actin core (0.29 *μ*m^2^ on glass and 0.22 *μ*m^2^ on 2 kPa gels) (Fig. S10 *B*), but detailed analysis showed that the number of podosomes with area >0.3 *μ*m^2^ decreased substantially on soft ECM (41% on glass vs. 15% on 2 kPa gels) (Fig. 5 *F*). Likewise, filopodia—thin membrane protrusions that cells use to probe the microenvironment—were formed by SSMs on all substrates but were shorter on soft ECM (Figs. 5 *G* and S10 *C*), with the proportion of filopodia >4 *μ*m in length shifting from 45% on glass to 13% on 2 kPa gels. Together, these results indicate that SSMs are mechanosensitive cells that adapt their morphology to changes in ECM rigidity.

### ECM rigidity alters the mobility of ICs

**Figure 6:**
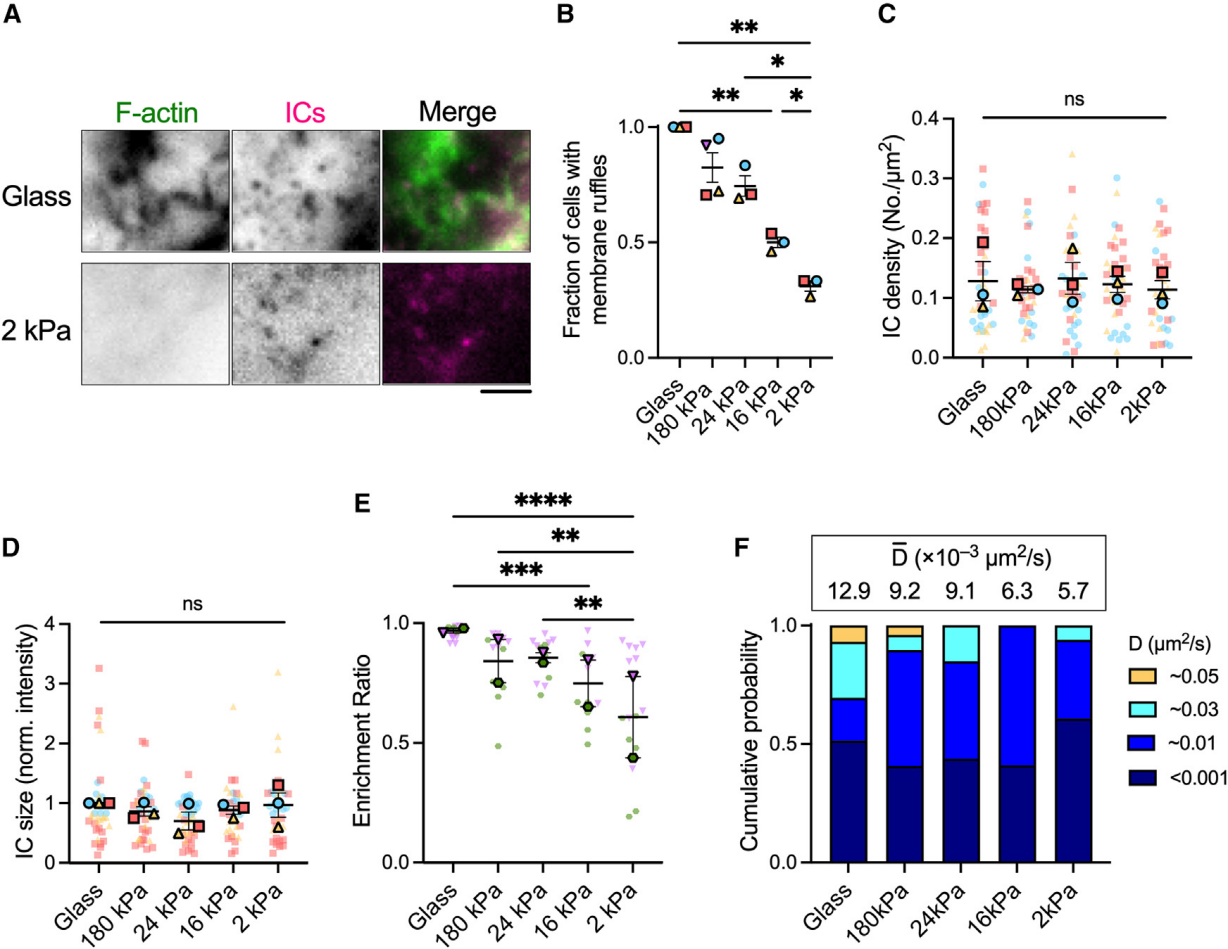
ECM rigidity alters membrane topography and IC mobility, but not the density or clustering of ICs. (*A*) Maximum intensity projection of three z-planes, totaling 0.6 *μ*m depth, at the dorsal membrane of SSMs cultured on collagen I-coated glass (*top row*) or 2 kPa polyacrylamide gel substrates (*bottom row*). Cy3B-labeled ICs (*magenta*) are present on both cell membranes, but actin-rich membrane ruffles (stained with phalloidin-AF488, *green*) are visible only in the cell cultured on glass. Scale bar, 2 *μ*m. (*B*) The fraction of cells on each substrate that have formed at least one membrane ruffle on the dorsal surface. *N* = 3 or 4 independent experiments. (*C* and *D*) Quantitation of the (*C*) surface density and (*D*) size of ICs presented by SSMs cultured on collagen I-coated glass or polyacrylamide gels of different stiffness. Each plain dot represents the mean value for all ICs on one cell and each outlined dot represents the mean value for all cells in one independent experiment (*n* = 8 to 19 cells per condition per experiment, *N* = 3 independent experiments). Data are color coded by experiment. Bars represent mean ± SEM. (*E*) Enrichment of ICs on actin-rich membrane ruffles on SSMs cultured on collagen I-coated gels of different stiffness (*n* = 5 to 14 cells per condition per experiment, *N* = 2 independent experiments). (*F*) Bar graphs showing the mean and weight fraction of each identified mobility state for ICs diffusing on cells cultured on collagen I-coated gels of different stiffness. Data are pooled from two independent experiments (2 kPa, 3095 trajectories from 7 cells; 16 kPa, 3020 trajectories from 4 cells; 24 kPa, 3100 trajectories from 5 cells; 180 kPa, 5340 trajectories from 8 cells; glass, 9160 trajectories from 7 cells.) The weighted mean of the diffusion constant for each condition, $\bar{D}$, is given atop each bar. ns, not significant. ^∗^*p* < 0.05, ^∗∗^*p* < 0.01, ^∗∗∗^*p* < 0.001, ^∗∗∗∗^*p* < 0.0001 (one-way ANOVA with Tukey’s multiple comparisons test). To see this figure in color, go online.

SSM sensing of ECM rigidity also affected the formation of membrane ruffles on the dorsal cell surface. SSM dorsal membranes were replete with actin-rich ruffles when cells were cultured on collagen I-coated glass (Fig. 6 *A*, Video S15) and devoid of them when cells were cultured on collagen I-coated 2 kPa gels (Fig. 6 *A*, Video S16). Analysis of membrane ruffle formation showed that the fraction of SSMs with at least one ruffle decreased with ECM rigidity, from 100% of cells on glass to only 31% of cells on 2 kPa gels (Fig. 6 *B*). This change in membrane topography did not impact the ability of SSMs to capture ICs, as the surface density (0.12 complexes/*μ*m^2^) (Fig. 6 *C*) and size (fluorescence intensity) (Fig. 6 *D*) of ICs were unchanged. The loss of membrane ruffles and retention of IC density resulted in a loss of spatial association between ICs and ruffles on the dorsal surface (Fig. 6 *E*).

Video S15. 3D rendering of confocal z-stack images of a phalloidin AF488-labeled SSM cultured on a collagen I-coated glass coverslip

Video S16. 3D rendering of confocal z-stack images of a phalloidin AF488-labeled SSM cultured on a collagen I-coated 2 kPa gel

Because we previously found that the actin cytoskeleton constrains the lateral motion of ICs (Figs. 3 and 4), we hypothesized that ECM stiffness-induced alterations in membrane ruffle formation would also impact IC mobility. We therefore tracked ICs on the surfaces of live SSMs cultured on collagen I-coated substrates of different stiffness and analyzed the trajectories using SMAUG. The weighted mean diffusion constant of ICs was 56% lower when SSMs were cultured on 2 kPa gels (0.0057 *μ*m^2^/s) compared with glass (0.0129 *μ*m^2^/s) (Fig. 6 *F*). Detailed analysis of the different mobility states showed that the fraction of immobile ICs ($D_{\text{immobile}}<0.001\;\mu m^{2}/s$) increased from 0.4 on glass to 0.6 on 2 kPa gels, and the fraction of ICs with $D\geq 0.03\;\mu m^{2}/s$ decreased from 0.31 on glass to 0.06 on 2 kPa gels. Thus, stiff collagen I matrices enhance membrane ruffling and increase the lateral mobility of ICs presented by SSMs.

### The accumulation of F-actin in SSM-B cell immune synapses is regulated by ECM rigidity

**Figure 7:**
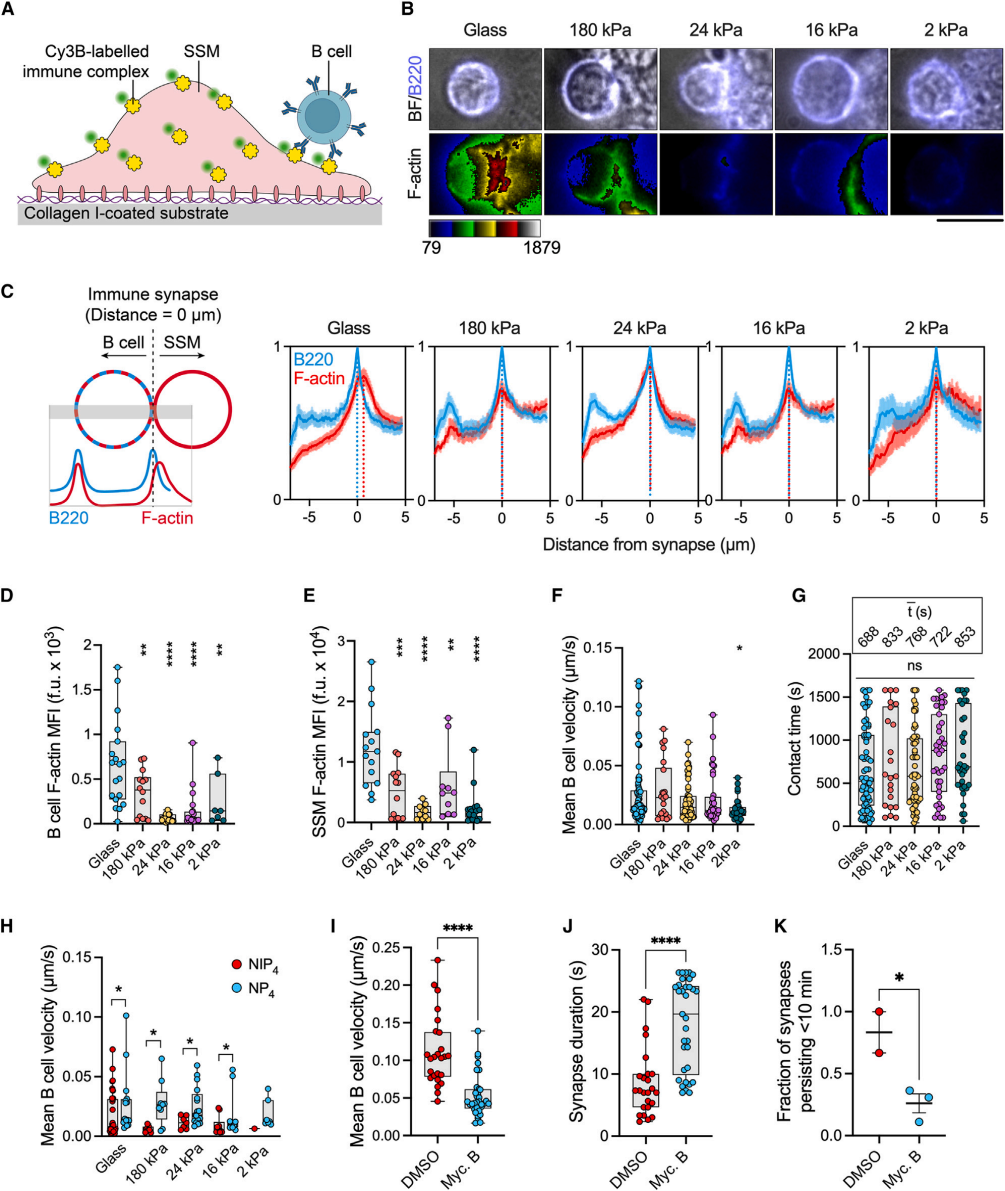
ECM rigidity influences F-actin accumulation in SSM-B cell immune synapses and regulates synapse duration. (*A*) Schematic representation of SSM-B cell immune synapses imaged by fluorescence microscopy. (*B*) Single z-plane images of primary mouse B cells contacting SSMs that are cultured on collagen I-coated gels of different stiffness. The SSMs present ICs that include anti-mouse Igκ F(abʹ)_2_ as surrogate antigen (Igκ-IC). The B cell membrane is labeled with anti-B220, and F-actin in both the B cells and SSMs is labeled with phalloidin. Scale bar, 5 *μ*m. (*C*) (*Left*) Schematic representation of the contact formed between a B cell and an SSM. The B cell membrane is labeled with B220 (*blue*) and supported by the cortical actin cytoskeleton (*red*). The SSM actin cytoskeleton is also labeled (*red*). Line intensity profiles through the gray boxed region show that on the left side of the B cell the B220 and F-actin signals overlap completely, while on the right side of the B cell the contribution of F-actin signal from the SSM shifts the F-actin maximum intensity to the right. (*Right*) Line intensity profiles of B220 (*blue*) and F-actin (*red*) in SSM-B cell contacts. SSMs cultured on glass polarize the actin cytoskeleton toward contacts with B cells, while SSMs cultured on gels do not. The solid line represents the mean value and the fills the standard errors of the mean. (*D* and *E*) Mean fluorescence intensity of F-actin (*D*) on the B cell side of the synapse and (*E*) on the SSM side of the synapse. The data in (*C–E*) are from *n* = 19 (glass), 15 (180 kPa), 20 (24 kPa), 21 (16 kPa), and 7 (2 kPa) synapses from two experiments. (*F*) The mean velocity and (*G*) contact time of B cells interacting with SSMs cultured on gels of different stiffness. The mean contact time, $\bar{t}$, is shown for each condition (*n* = 22 to 77 B cells per condition, pooled from two experiments). (*H*) Mean velocity of B1-8 mouse B cells interacting with SSMs presenting ICs that contain either high-affinity NIP_4_ (NIP_4_-IC) or low-affinity NP_4_ antigen (NP_4_-IC). (*I–K*) Synapse duration is regulated by the SSM actin cytoskeleton. Treating SSMs with mycalolide B (*I*) reduces B cell velocity, (*J*) increases the immune synapse duration, and (*K*) inhibits B cell dissociation from the SSM. Boxes indicate the interquartile range (first quartile to third quartile), the line indicates the median value, and the whiskers extend to the minimum and maximum points. ns, not significant. ^∗^*p* < 0.05, ^∗∗^*p* < 0.01, ^∗∗∗∗^*p* < 0.0001, Glass versus gels (*D–F*); one-way ANOVA with Dunnett’s multiple comparison’s test); NIP_4_ versus NP_4_ (*H*); unpaired *t*-test); DMSO versus Myc. B (*I–K*, unpaired *t*-test). To see this figure in color, go online.

We next wondered about the capacity of SSMs to form contacts with B cells and stimulate B cell activation, and whether ECM rigidity would influence these functional behaviors. To investigate this question, we cultured SSMs on collagen I-coated glass and gel substrates and loaded them with fluorescent ICs containing anti-mouse Igκ F(abʹ)_2_ as surrogate antigen (Igκ-IC). We then added naive B cells from C57BL/6 mice and allowed them to interact with SSMs for 20 min at 37°C before imaging (Fig. 7 *A*). B cells formed contacts (immune synapses) with SSMs that were adhered to all substrates (Fig. 7 *B*). The images showed that both B cells and SSMs accumulated F-actin in the synapse, and the extent of actin remodeling was influenced by ECM rigidity (Fig. 7 *B*). We used a line profile analysis of fluorescence intensities to quantify the enrichment of F-actin on both sides of the immune synapse, using B220 to define the B cell boundary. On the distal side of the B cell (i.e., the side facing away from the SSM), the B220 and phalloidin signals peaked together, while on the synapse side of the B cell, the phalloidin signal skewed toward the SSM, reflecting the contribution of the SSM actin cytoskeleton (Fig. 7 *C* schematic). The enrichment of F-actin on both sides of the synapse was influenced by ECM rigidity. Synaptic accumulation of F-actin was greatest for both cells when SSMs were cultured on glass, and decreased in both cells as the substrate became softer (Fig. 7, *C–E*).

To determine whether the alteration in actin remodeling impacted other aspects of the immune synapse, we performed live-cell imaging of SSM-B cell interactions (Video S17). These data showed that B cells formed transient contacts with SSMs and often migrated along their surfaces. As the ECM became softer, B cells migrated with a lower velocity (Fig. 7 *F*) and formed more durable contacts with SSMs (Fig. 7 *G*), although the latter trend did not reach statistical significance. Nevertheless, the results overall support the conclusion that ECM-SSM tension influences the stability of synapses formed between SSMs and B cells. To determine whether synapse stability was dependent on BCR signaling, we loaded SSMs with ICs containing either NIP_4_ or NP_4_ antigen (NIP_4_-IC or NP_4_-IC) to stimulate naive B1-8 B cells, which bind these two antigens with a 10-fold difference in affinity ($K_{d}^{\text{NIP}}=0.1\;\mu M$ and $K_{d}^{\text{NP}}=1\;\mu M$) (8). B cells engaging high-affinity NIP_4_-IC formed more durable contacts than B cells engaging low-affinity NP_4_-IC (Fig. 7 *H*). BCR signaling thus influences the stability of B cell interactions with SSMs, in agreement with studies showing that the probability of synapse formation increases with BCR-antigen affinity (7,59) and that antigen recognition encourages prolonged B cell retention in the lymph node subcapsular sinus region (19).

Video S17. Time-lapse images of B cells contacting SSMs cultured on collagen I-coated substrates Images were acquired at 11.5 × magnification, with 100 ms exposures taken every 20 s, at 37°C. B cells are loaded with Cal-520 (*green*) and SSMs are labeled with Cy3B-ICs (*magenta*). Scale bar, 5 *μ*m.

We hypothesized that the effects of ECM rigidity on B cell velocity and contact duration were due to reduced F-actin content in SSMs. To test this hypothesis, we permanently depleted the SSM actin cytoskeleton with mycalolide B, removed the drug from the wells, added untreated primary B cells, and imaged the cells live. The loss of SSM F-actin significantly reduced B cell velocity (Fig. 7 *I*) and increased SSM-B cell contact time (Fig. 7, *J* and *K*), indicating that an intact SSM actin cytoskeleton was required for the dissolution of the SSM-B cell immune synapse. Taken together, our data suggest that SSMs respond to contacts with B cells by remodeling their actin cytoskeleton, and that ECM-SSM force transmission alters SSM-B cell synapse duration by affecting actin polymerization in both cells.

### SSM sensing of ECM rigidity does not impact B cell antigen internalization or NF-κB activation

**Figure 8:**
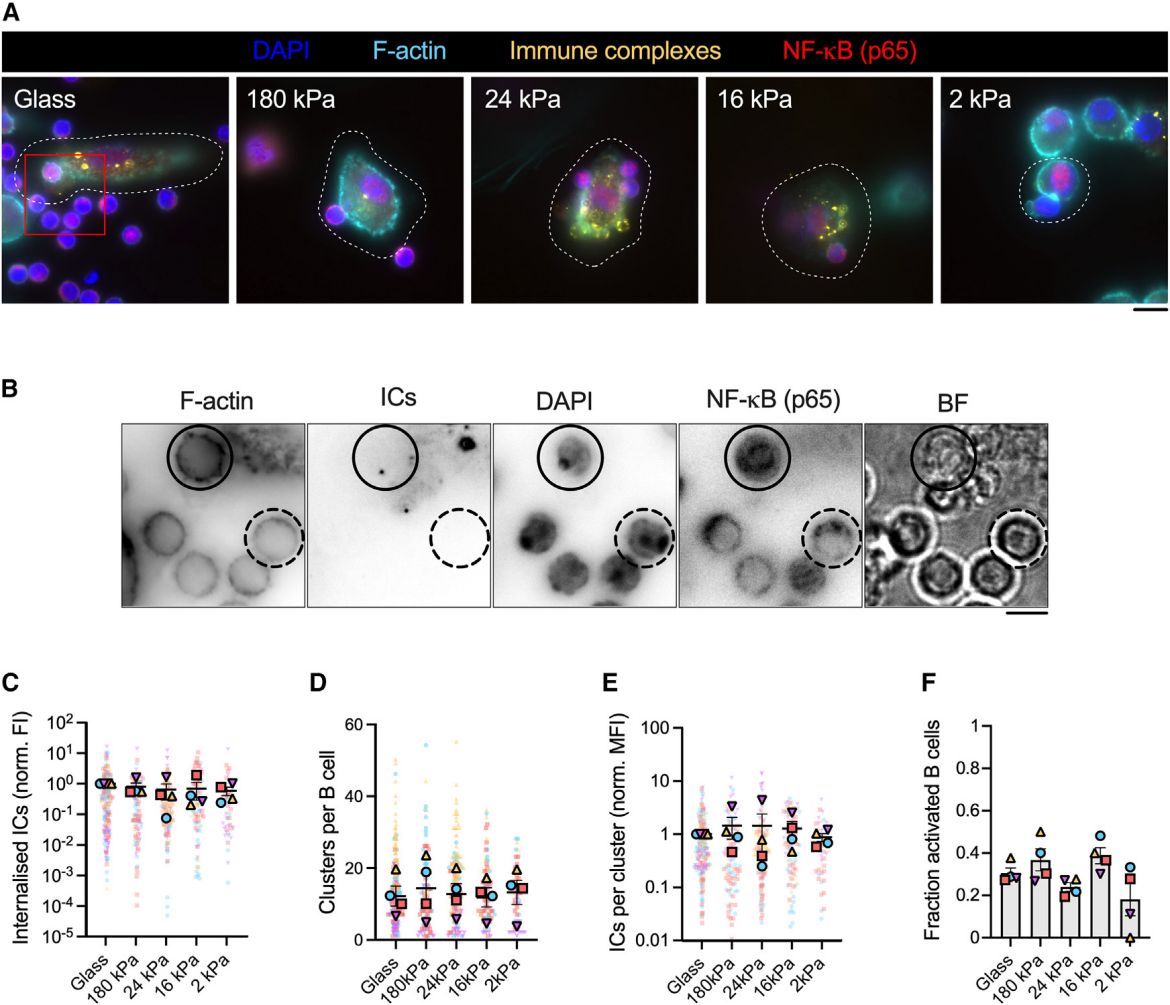
SSM sensing of ECM rigidity does not impact B cell IC internalization or NF-κB activation. (*A*) Images of primary mouse B cells interacting with SSMs on collagen I-coated substrates of different stiffness. The images are single z-planes several *μ*m above the substrate. F-actin is visualized by phalloidin-AF488 and ICs contain Cy3B-labeled anti-Igκ F(abʹ)_2_ as surrogate antigen (Igκ-IC). SSM boundaries are outlined with dotted white lines. Scale bar, 10 *μ*m. (*B*) Zoomed in region highlighted by the red box in (*A*) showing B cells stained with phalloidin, DAPI, and anti-NF-κB (p65). The B cell outlined with a solid circle has acquired antigen from an SSM and has translocated p65 to the nucleus, while the B cell outlined with a dotted circle has not acquired antigen nor translocated p65. Scale bar, 5 *μ*m. (*C–E*) Features of B cell Igκ-IC internalization, including (*C*) the total amount of Igκ-IC internalized per cell, (*D*) the number of internalized Igκ-IC clusters per cell, and (*E*) the average amount of Igκ-IC per cluster. (*F*) The fraction of B cells that activate after encountering SSMs adhered to substrates of different stiffness, assessed by p65 translocation to the nucleus. p65 translocation was measured for each B cell by calculating the ratio of nuclear to cytosolic anti-NF-κB (p65) intensity, $I_{\text{nuc}}/I_{\text{cyt}}$. B cells with a $I_{\text{nuc}}/I_{\text{cyt}}$ ratio greater than 1 were defined as activated. For the data in (*C–F*), each plain dot represents one cell and each outlined dot represents the mean value for one independent experiment (*n* = 50 to 235 cells per condition, *N* = 4 independent experiments). Data are color coded by experiment. Bars represent mean ± SEM. To see this figure in color, go online.

In vitro studies of B cells interacting with antigen-coated substrates have established that actin remodeling is essential for robust BCR signaling and internalization of antigen. The role of APC mechanics in regulating these processes is less well understood. We therefore measured B cell internalization of Igκ-ICs from SSMs cultured on collagen I-coated gels of different stiffness (Fig. 8, *A* and *B*). Wild-type (C57BL/6) naive B cells internalized similar amounts of Igκ-IC from SSMs regardless of ECM rigidity—the total amount of Igκ-IC, number of Igκ-IC clusters, and Igκ-IC content per cluster were the same across all substrates (Fig. 8, *C–E*). Consistently, using NF-κB (p65) nuclear translocation as a downstream readout of transcriptional activation, we found that B cells had an equal probability of activating regardless of ECM rigidity (Fig. 8 *F*). Although the mechanism remains unclear, we speculate that the effects of ECM rigidity on SSMs—altered actin remodeling (Figs. 5 and 8) and IC mobility (Fig. 6)—may coordinate to ensure consistent B cell IC internalization and NF-κB activation in different mechanical environments.

## Discussion

Our study shows that physical properties of SSM-B cell immune synapses are shaped by the mechanical microenvironment. SSMs use actin-based membrane protrusions to control the spatial organization, mobility, and topography of ICs presented on their surfaces. These properties have all been described from in vitro studies to influence early events in B cell activation (12,13,16). A key finding from our paper is that these physical aspects of IC presentation by SSMs are not static but rather dynamic properties that are controlled by the actin cytoskeleton and modulated by ECM stiffness. Our data indicate that mechanotransduction at ECM-SSM interfaces regulates SSM-B cell contact duration and actin remodeling in both cells. Despite these differences, B cell internalization of ICs and activation of NF-κB were not impacted, suggesting that these processes may be mechanically insulated from changes in the environment.

In the lymph node, B cells migrate to the subcapsular region to sample SSM surfaces for antigens (21). Most B cells spend <5 min in contact with SSMs (19), so they must be efficient in their search. B cells rapidly scan their BCRs over antigen-presenting surfaces using actin-based membrane protrusions (60,61,62,63), which is a general mechanism employed by lymphocytes to locate antigens (64). We show here that SSMs, too, employ active surface topography to direct IC motion, suggesting that SSMs may survey B cell membranes in return to facilitate encounters between ICs and BCRs.

Constraining IC motion may be another mechanism by which SSMs exert control over the B cell response. In vitro studies have shown that B cells interacting with monovalent anti-Ig Fab molecules on fluid planar lipid bilayers ($D_{\text{Fab}}\approx 4\;\mu m^{2}/s$) assemble large BCR-antigen microclusters with robust phosphotyrosine signaling (13). B cells encountering the same Fab molecules on glass ($D_{\text{Fab}}=0\;\mu m^{2}/s$) form smaller clusters and have an attenuated response (13). In this paper, we show that neither scenario is an accurate representation of antigen presentation by SSMs. Instead, we show that ICs on SSM surfaces flit between immobile ($D_{\text{IC}}<0.001\;\mu m^{2}/s$) and moderately mobile ($D_{\text{IC}}\approx 0.03-0.05\;\mu m^{2}/s$) states in a way that depends upon actin. These diffusion constants match those of BCRs ($D_{\text{IgM}-\text{BCR}}\approx 0.03\;\mu m^{2}/s$ and $D_{\text{IgD}-\text{BCR}}\approx 0.003\;\mu m^{2}/s$) (65), suggesting that SSMs may resist the lateral transport of BCR-antigen complexes to inhibit microcluster growth—a mechanism that has been linked to improved discrimination of antigen affinities (11). While lowering ECM stiffness caused modest changes in IC mobility—$D_{\text{glass}}\approx 0.013\;\mu m^{2}/s$ and $D_{2\text{kPa}}\approx 0.006\;\mu m^{2}/s$—it did not have a statistically significant effect on B cell NF-κB activation. This result may indicate that B cells are not sensitive to antigen mobility changes in this range, although other possibilities cannot be ruled out. Our study used multivalent antigens, which are more efficient than monovalent antigens at triggering BCR activation (66) and may therefore lessen the sensitivity to small changes in mobility. B cells in our experiments could also engage coreceptors that decrease the threshold of BCR-dependent stimulation, including LFA-1 and VLA-4 binding to ICAM-1 and VCAM-1, respectively, on the SSM surface (59,67); and CD19-associated CD21 binding to C3d on the IC (68,69). Adopting new in vitro platforms that express different combinations and concentrations of ligands to better mimic SSM surfaces will be important for understanding the relative contributions of antigen valency, antigen mobility, and accessory receptor engagement on the B cell response (70).

Our data indicate that the SSM actin cytoskeleton controls the stability of contacts with B cells. Similar observations have been made for interactions between APCs and T cells (71,72), suggesting that activity of the APC actin cytoskeleton might be generally required for regulating synapse duration. It has been proposed that the dissolution of the synapse relies on the APC cytoskeleton transmitting mechanical force to antigen and integrin receptors to modulate their signaling activity (72). For this process to occur, the APC would need to be sufficiently rigid to counteract the mechanical load on receptor-ligand bonds. We have observed that softening the ECM leads to prolonged SSM-B cell contacts and impairs F-actin assembly on both sides of the synapse, which is consistent with reduced force transmission between the two cells. Mechanoreciprocity has been observed in various cellular systems ranging from epithelial cell adhesion (29) to collective cell migration (73), so it is conceivable that the magnitude of forces exerted at the ECM-SSM interface is reflected in SSM-B cell contacts. However, we cannot exclude the possibility that changes in ECM stiffness may also impact the expression levels of integrin ligands that regulate cell-cell adhesion (74).

B cells use pulling forces to extract antigens from APCs for internalization (15). Force application makes B cells responsive to APC stiffness—B cell antigen internalization is more efficient when antigens are linked to soft APCs, and less efficient but more stringent when antigens are linked to stiff APCs (75). Our experiments reveal that the efficiency of IC internalization by B cells is not influenced by ECM-SSM tension. This finding suggests that SSMs do not adjust their stiffness to match their surroundings. While direct measurements of SSMs adhered to different matrices are needed for confirmation, it is worth noting that insensitivity of cortical cell tension to substrate mechanics is a common behavior observed in various cell types (71,76,77). It is also possible that SSMs locally increase stiffness in response to interactions with B cells (78). However, the reciprocal recruitment of F-actin to the synapse in both B cells and SSMs implies that the relative stiffness of BCR-antigen and antigen-APC bonds remains constant, maintaining a consistent likelihood of antigen extraction (79). Nevertheless, a B cell that acquires antigen but is slow to detach from SSMs would likely have dysregulated responses due to sustained BCR stimulation, which has been associated with a hyperactivated state (80,81,82) and an inability to recruit timely T cell help (83,84).

In summary, we have established a role for the SSM actin cytoskeleton in coupling ECM mechanics to immune synapses formed with B cells. We suggest that the ECM may play a role in orchestrating B cell activation through its effects on antigen presentation by SSMs.

## Author contributions

M.I. designed and performed the experiments, analyzed the data, and helped prepare the manuscript. A.T.B. performed flow cytometry experiments, analyzed the data, and provided advice. C.C. performed flow cytometry experiments and analyzed the data. H.C.W.M. developed image analysis pipelines. M.G. and F.A. analyzed imaging data. R.K. analyzed flow cytometry data and provided LifeAct-GFP mice. A.P.C. designed the experiments and provided reagents and advice. K.M.S. designed the experiments, supervised the research, and wrote the manuscript. All authors contributed to revising and editing the manuscript.

## Supporting material

Document S1. Figures S1–S10 and Tables S1–S3

Document S2. Article plus supporting material
