# Supplementary material for: Extracellular matrix rigidity modulates physical properties of subcapsular sinus macrophage-B cell immune synapses
Source: Biophys J. 2023 Oct 14;123(15):2282–300. doi: 10.1016/j.bpj.2023.10.010 (PMC11331050; doi:10.1016/j.bpj.2023.10.010)
Supplement: Document S1. Figures S1–S10 and Tables S1–S3 [file mmc1.pdf]

Biophysical Journal, Volume *123*

Supplemental information

Extracellular matrix rigidity modulates physical properties of subcapsular sinus macrophage-B cell immune synapses

Maro Iliopoulou, Anna T. Bajur, Hannah C.W. McArthur, Michael Gabai, Carl Coyle, Favour Ajao, Robert Köchl, Andrew P. Cope, and Katelyn M. Spillane

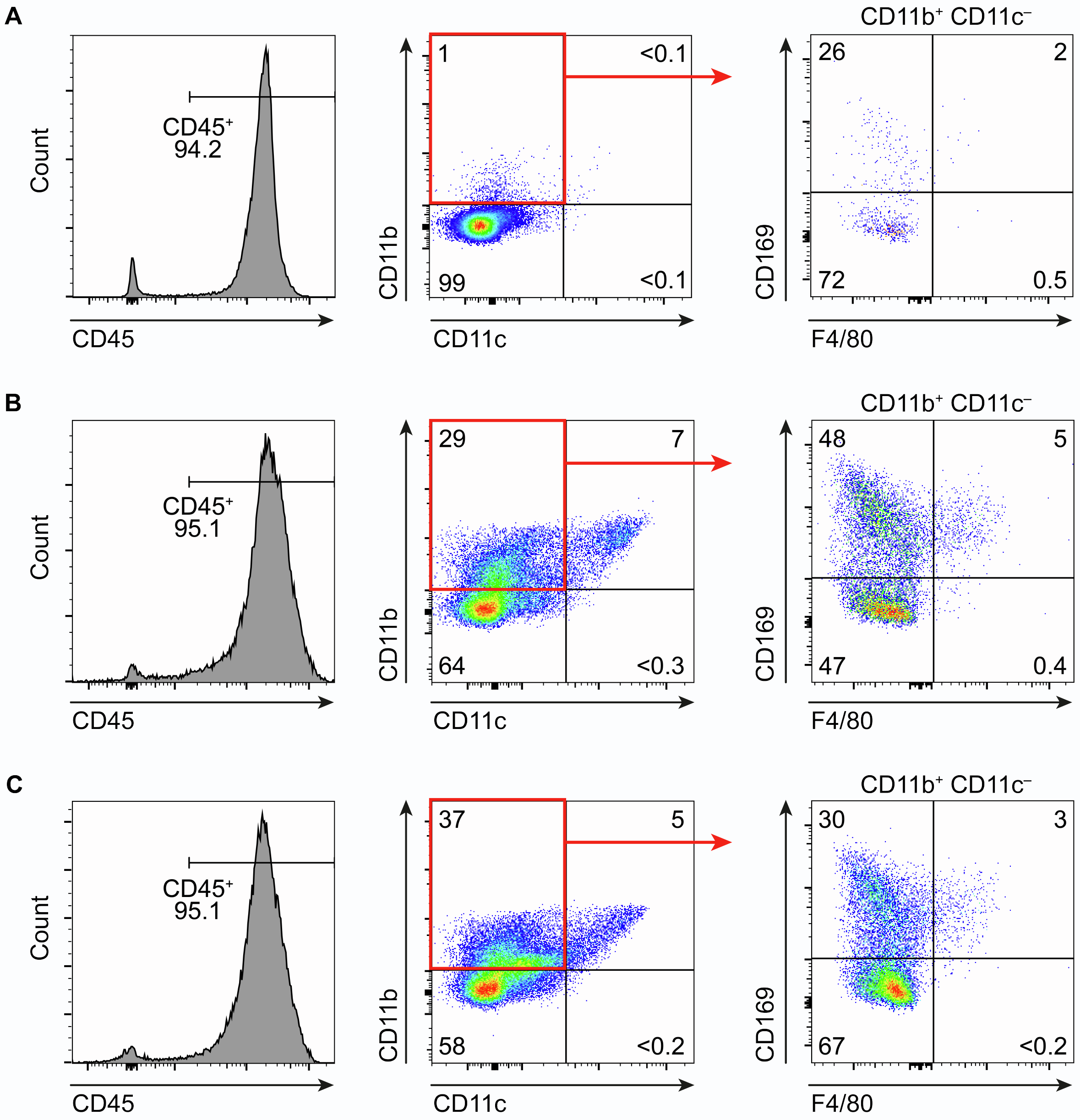

Figure S1 Isolation and identification of SSMs. Pseudocolour flow cytometry plots of live single-cell suspensions from (A) whole lymph nodes, and (B,C) enzyme-digested lymph nodes positively enriched by (B) an FDC-specific antibody (rat IgG2c, κ anti-mouse FDC-M1) or (C) a rat IgG2c, κ isotype control antibody, both complexed with a biotinylated mouse IgG2a anti-rat Ig κ secondary antibody and captured by anti-biotin microbeads. Cells were stained with monoclonal antibodies to CD45, CD11b, CD11c, CD169, and F4/80 (see Table S1). The proportions of all cell populations are indicated on the plots. Data are representative of three experiments.

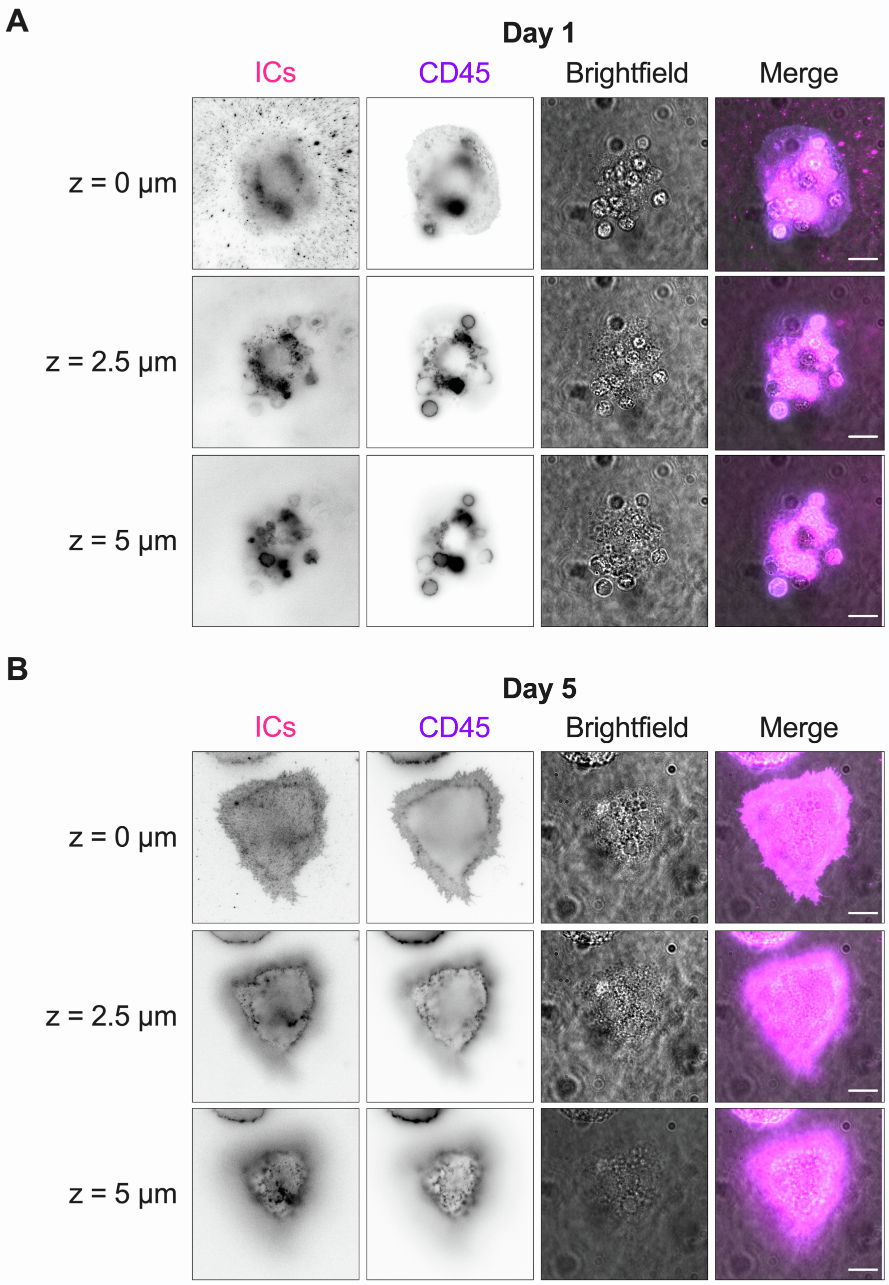

Figure S2 SSM cultures contain lymphocytes on Day 1 but not Day 5. IC (IC; Cy3B-labelled) and anti-CD45 staining of SSMs cultured for (A) one or (B) five days on collagen I-coated glass. The single-plane images at 0, 2.5, and 5 μm above the coverslip were taken from z-stack images. On Day 1, many small, round lymphocytes (IC^{+} $CD45^{+}$) can be seen adhering to the SSMs. The presence of lymphocytes prevents the characterisation of SSM morphology, actin structures, and IC distribution. By Day 5, the lymphocytes have died and can be washed away.

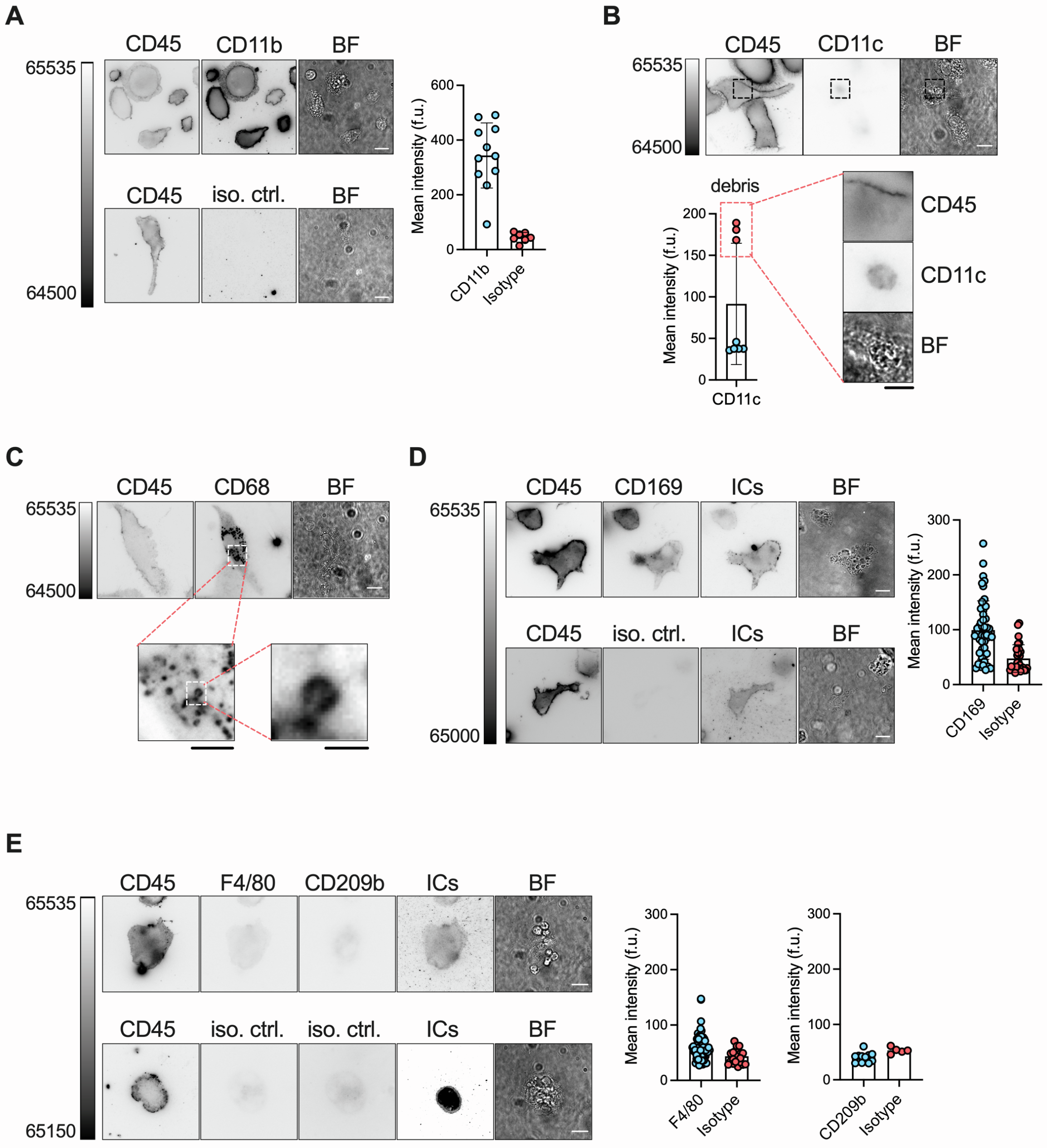

Figure S3 Identification of SSMs in culture with multi-colour fluorescence microscopy. SSMs were enriched from lymph node single-cell suspensions, cultured in vitro on collagen I-coated glass coverslips, labelled with fluorescent ICs (ICs), and stained with monoclonal antibodies to CD45, CD11b, CD11c, CD68, CD169, F4/80, and CD209b. Immunofluorescence imaging and quantitation confirmed that the cells are (A) CD45^{+} CD11b^{+}, (B) CD11c^{-}, (C) CD68^{+}, and (D) CD169^{+}. As expected, antibodies against CD45, CD11b, and CD169 specifically label the plasma membrane, while anti-CD68 labels the membranes of intracellular vesicles. Though several cells had relatively high mean intensity values of CD11c staining, close inspection of the images reveals that this signal is due to cell debris and not specific staining of the cell membrane. (E) The cells expressed no or very low amounts of the medullary macrophage markers F4/80 and CD209b. n = 5 to 48 cells collated from 2 biological replicates. Scale bars: 10 µm. Inset scale bars: (B) 5 µm; (C) left inset: 5 µm, right inset: 1 µm.

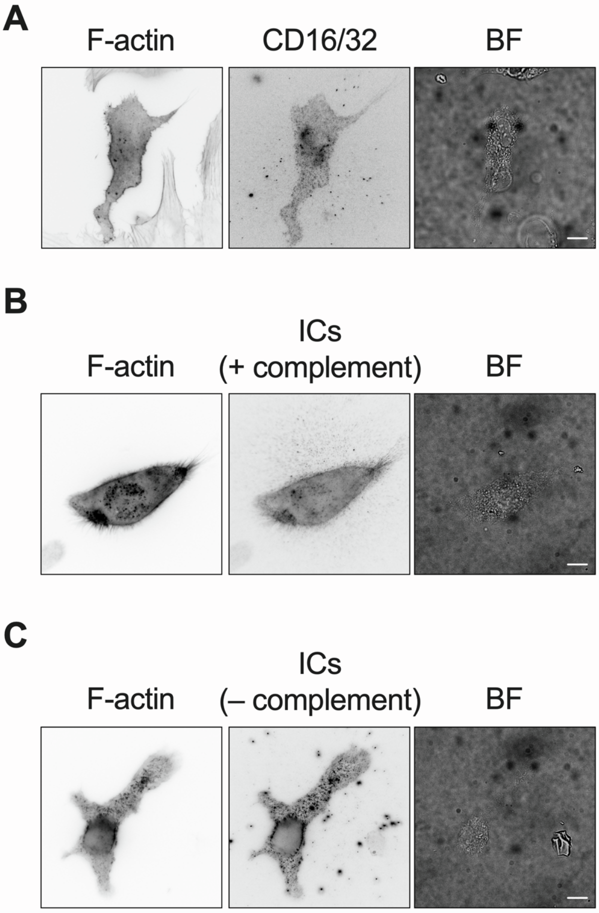

Figure S4 SSMs use FcγRs to present ICs. (A) Immunofluorescence staining with anti-CD16/32 (FcγRIII/FcγRII). Both SSMs (F-actin$^+$ CD16/32$^+$) and non-SSMs (F-actin$^+$ CD16/32$^-$) are visible in this image. (B, C) The ability of SSMs to capture and present ICs does not depend upon complement. IgG antibody complexes are captured equally well when (B) they are (+) and (C) are not (–) fixed with complement. Scale bars: 10 μm.

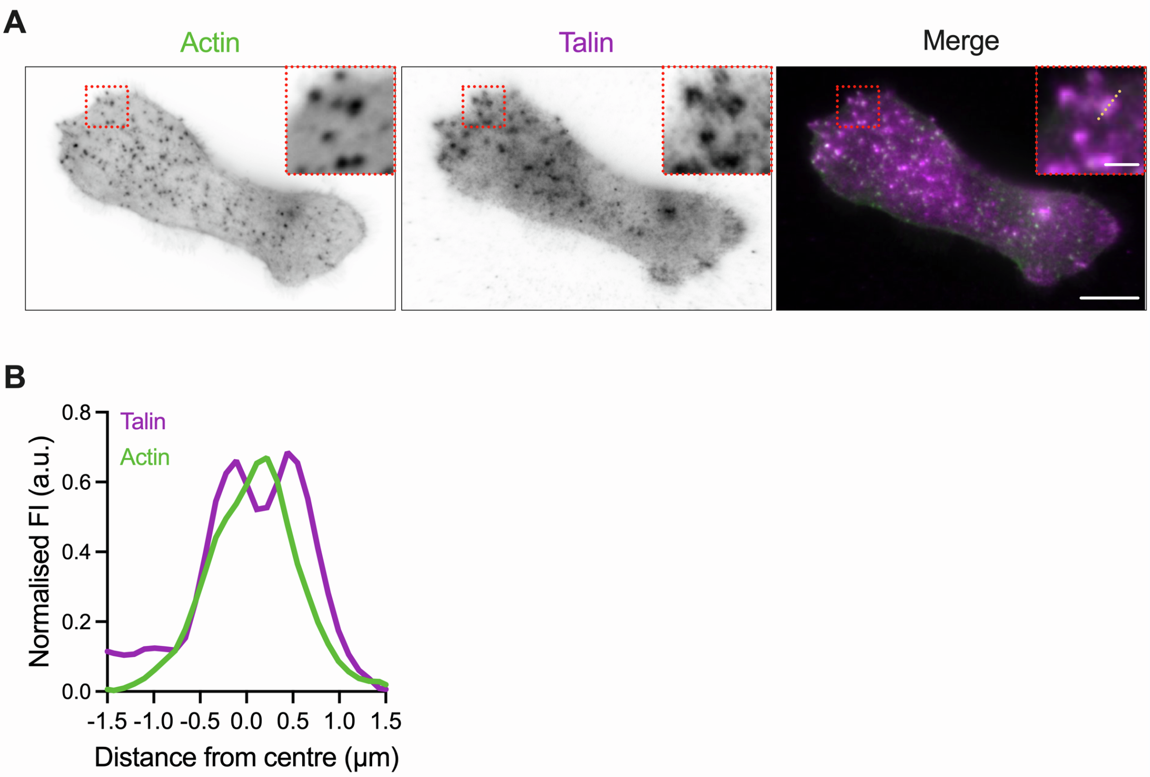

Figure S5 Characterisation of SSM podosomes. (A) TIRF microscopy images of an SSM stained with phalloidin-AF488 (green) and anti-talin (magenta). The insets depict a few individual podosomes. Scale bar: 10 µm; inset scale bar: 2 µm. (B) The fluorescence profile of actin and talin along the yellow dotted line in the Merge inset from (A).

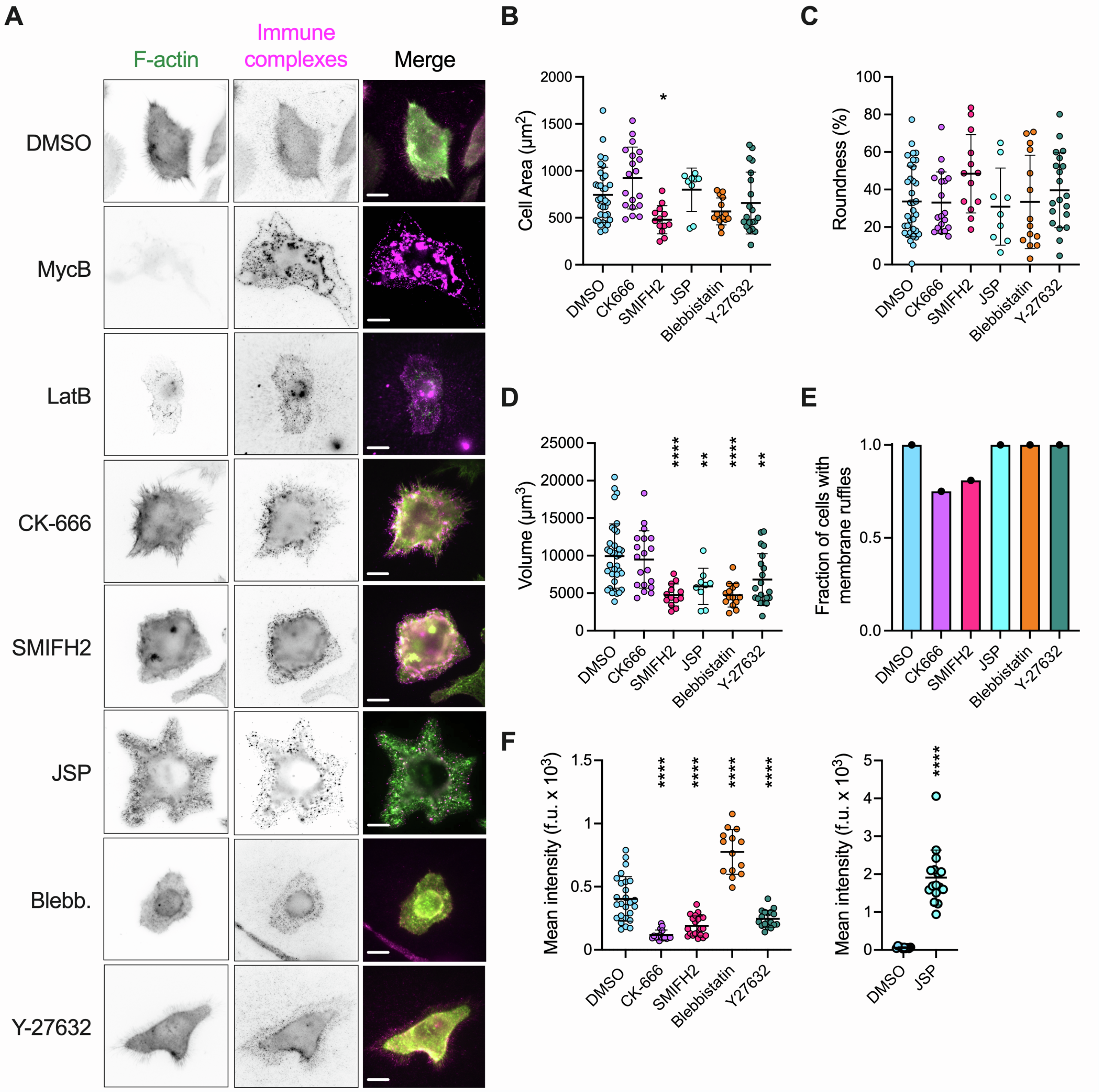

Figure S6 Inhibition of SSM actin dynamics and contractility. (A) F-actin (phalloidin-AF488) and IC (Cy3B-labelled) staining of SSMs treated with DMSO or inhibitors of the following targets: Arp2/3 (CK-666), formins (SMIFH2), Rho-associated kinase (Y-27632), myosin II (blebbistatin), actin disassembly (fluorogenic form of jasplakinolide (JSP), SiR-Actin), and actin assembly (mycalolide B and latrunculin B). Inhibitor concentrations are listed in Table S2. Scale bars: 10 µm. (B-D) Influence of the inhibitor treatments on cells as assessed by (B) spread area (µm²), (C) roundness (%), (D) volume (µm³), (E) the fraction of cells with at least one dorsal membrane ruffle, and (F) mean fluorescence intensity of F-actin. The two plots are generated from cells imaged in different experiments. Each dot in (B-D, F) represents one cell (n = 9 to 35 cells per condition, from one experiment). Bars represent mean ± SD. *$p<0.05$, **$p<0.01$, ****$p<0.0001$, DMSO-treated cells versus inhibitor-treated cells (one-way ANOVA for B-D and F (left); unpaired t-test for F (right)).

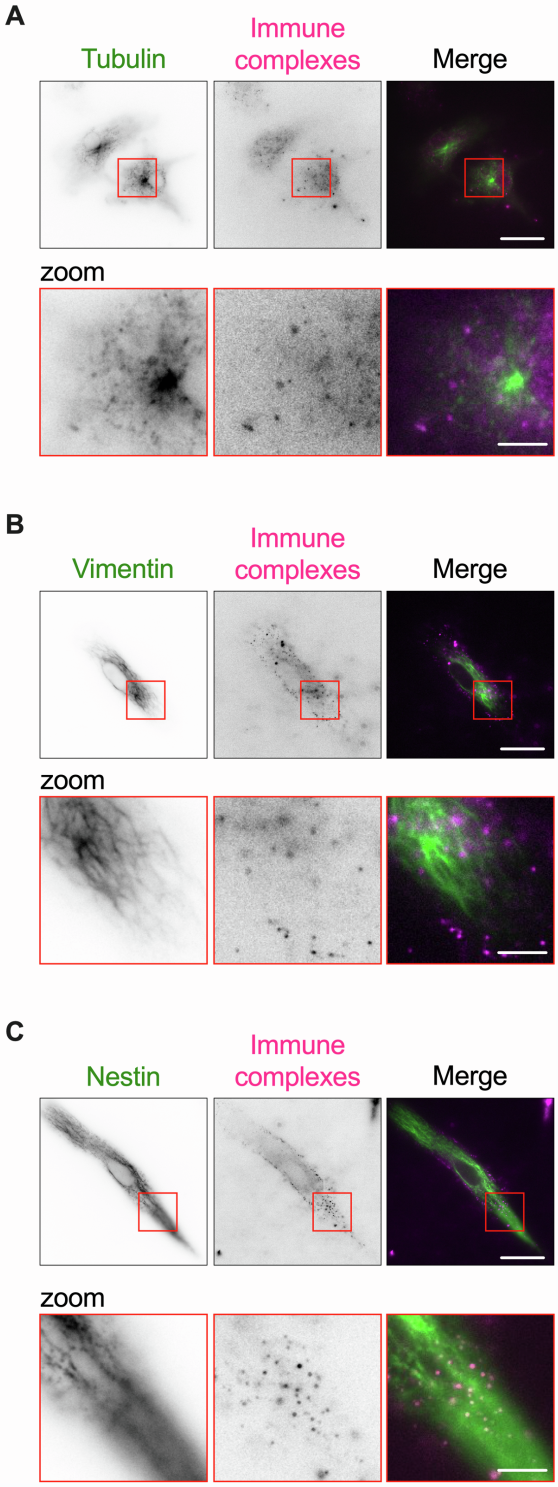

Figure S7 ICs presented by SSMs do not associate with microtubules or intermediate filaments. SSMs were cultured on collagen I-coated glass, labelled with Cy3B-ICs, and stained for (A) tubulin (z-position: 2 µm), (B) vimentin (z-position: 2.4 µm), and (C) nestin (z-position: 3.6 µm). Scale bars: 20 µm. Zoom scale bars: 5 µm.

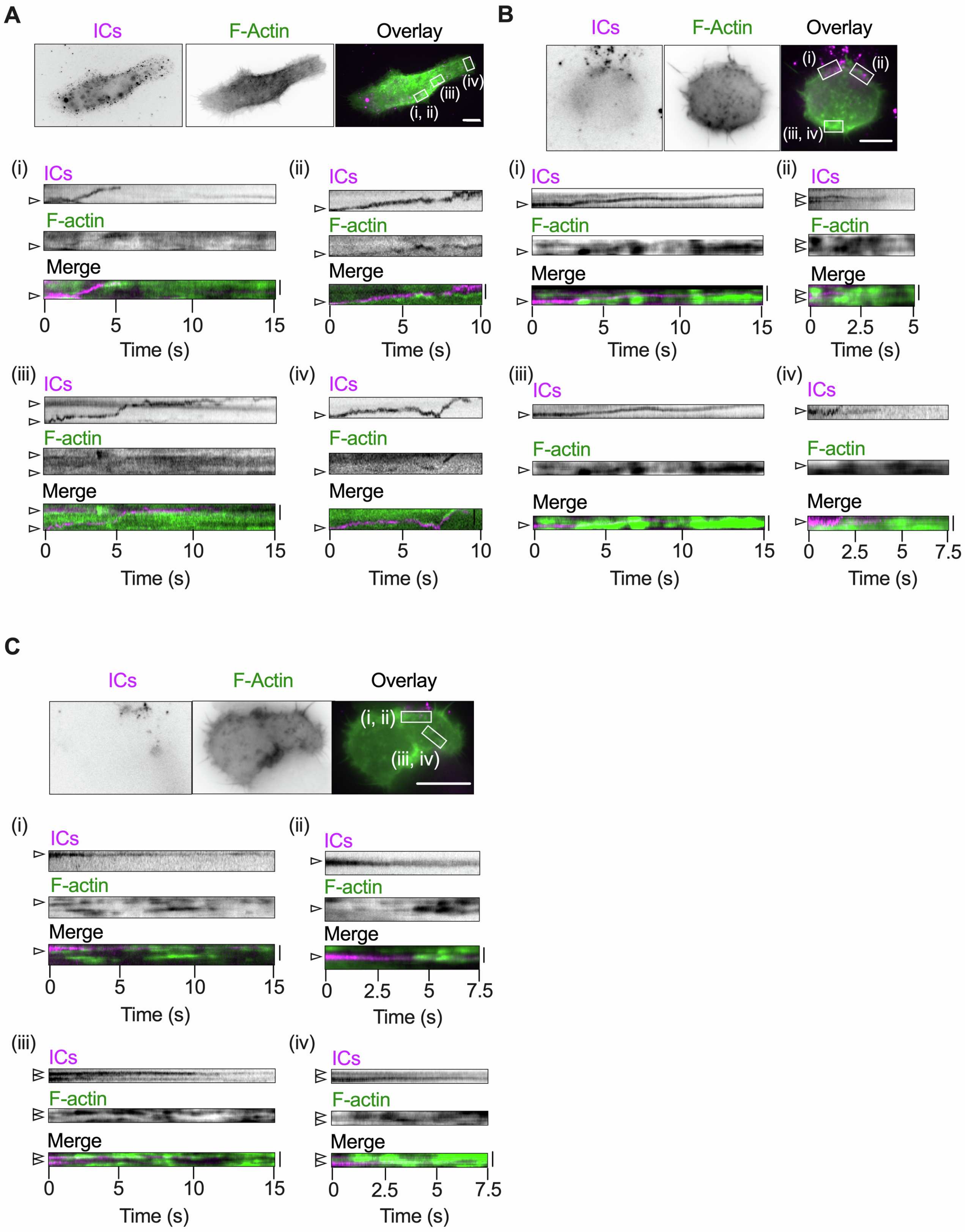

Figure S8 Kymographic analysis of F-actin and IC motion. (A-C) Kymographs representing the motion of ICs and F-actin from the indicated boxed regions. Arrow heads show the starting positions of ICs in each kymograph. Horizontal scale bars: 10 μm. Vertical scale bars: 2 μm.

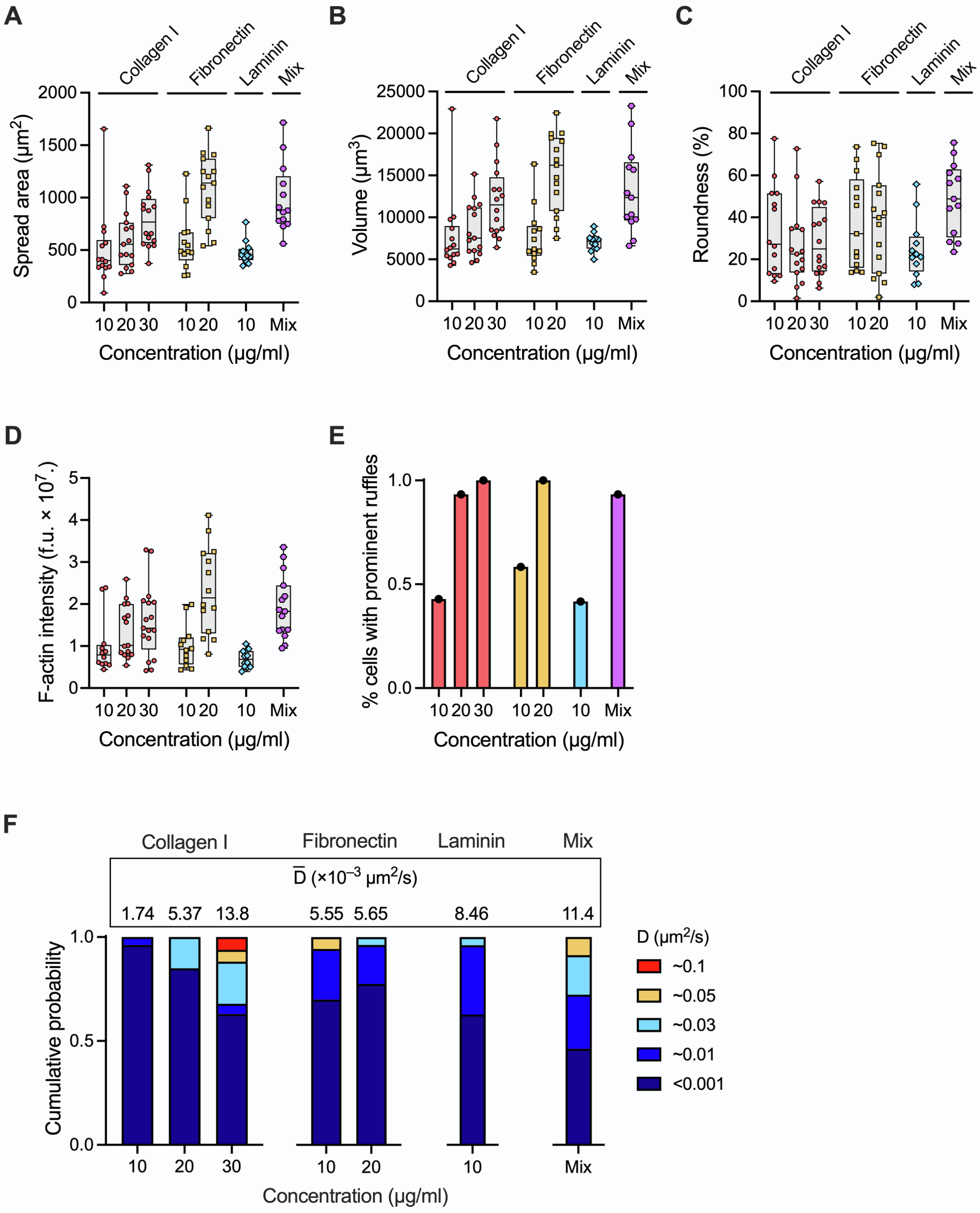

Figure S9 ECM ligand density and identity alters SSM morphology and IC mobility. (A-C) Cells change their morphology in response to ECM ligand composition as assessed by (A) spread area (μm^2), (B) volume (μm^3), and (C) roundness (%). The 'Mix' condition is a mixture of collagen I (10 μg/ml), fibronectin (20 μg/ml), and laminin (0.5 μg/ml) that we used as an approximation of their relative abundance in lymph node ECM. (D) Integrated fluorescence intensity of F-actin (phalloidin) at the dorsal cell membrane. Higher intensity values indicate more prominent F-actin structures. Each dot in (A-D) represents one cell (n = 12 to 16 cells per condition, from one experiment). Boxes indicate the interquartile range (first quartile to third quartile), the line indicates the median value, and the whiskers extend to the minimum and maximum points. (E) The fraction of cells on each ECM that have formed prominent dorsal membrane ruffles. (F) Bar graphs showing the mean weight fraction, π, of each mobility state identified by SMAUG analysis for ICs diffusing on SSMs adhered to glass coverslips coated with collagen I, fibronectin, or laminin at the indicated concentrations. The plots were constructed from the trajectories of >1500 ICs (n = 2-3 cells per condition, N = 1 experiment). The weighted mean of the diffusion constant for each condition, $\overline{\mathrm{D}}$, is given atop each bar.

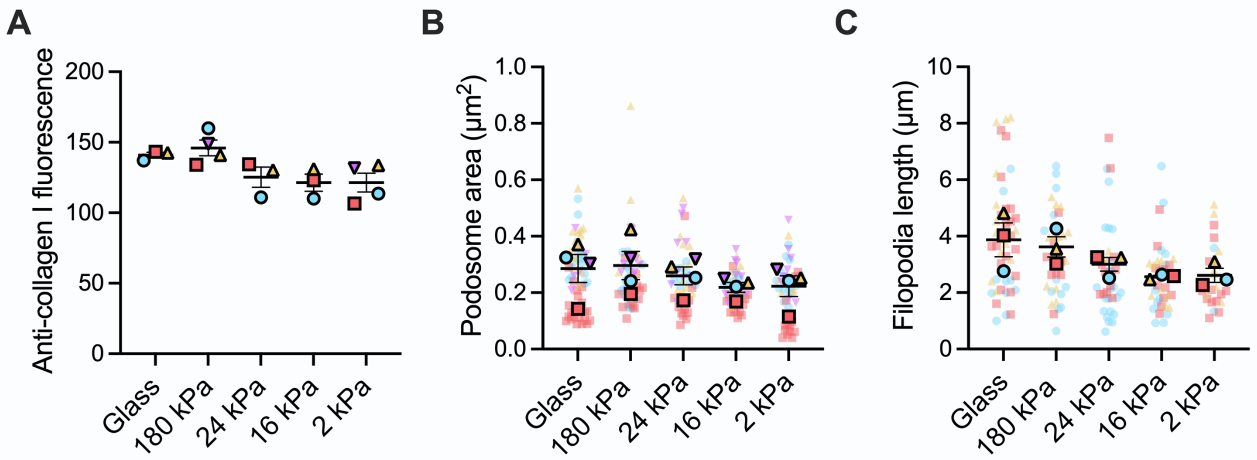

Figure S10 Characterising polyacrylamide gels and cell morphology. (A) Collagen I-coated glass and gel substrates were stained with a fluorescent anti-collagen I antibody and imaged with epifluorescence microscopy. The fluorescence intensity was the same across gels of different rigidity, indicating a consistent surface density of collagen I. Data are the mean fluorescence intensities from 3 or 4 substrates of each stiffness. (B) Podosome areas and (C) filopodia lengths for SSMs cultured on collagen I-coated substrates of different stiffness. Each plain dot represents one cell and each outlined dot represents the mean value for one independent experiment. n = 2 to 23 cells per condition per experiment, N = 3 or 4 independent experiments. Bars represent mean ± SEM.

Antibody	Clone	Final conc.	Supplier	Cat. No.	Assay
CD45-AF647	30-F11	0.05 µg/ml	Biolegend	103123	Flow cytometry/ Imaging
CD169-FITC	3D6.112	0.05 µg/ml	Biolegend	142405	Flow cytometry/ Imaging
CD11b-PE/Cy7	M1/70	0.04 µg/ml	eBioscience	25-01112-81	Flow cytometry
CD11c-APC/Cy7	N418	0.04 µg/ml	Biolegend	117323	Flow cytometry
F4/80-Pacific Blue	BM8	0.05 µg/ml	Biolegend	123123	Flow cytometry/ Imaging
CD45-APC/Cy7	30-F11	0.05 µg/ml	Biolegend	103115	Flow cytometry
Rat IgG2a-FITC iso ctrl	eBR2a	0.05 µg/ml	eBioscience	11-4321-71	Flow cytometry/ Imaging
Rat IgG2a-Pacific Blue iso ctrl	RTK2758	0.05 µg/ml	Biolegend	400527	Flow cytometry/ Imaging
Rat IgG2a-APC/Cy7 iso ctrl	RTK2758	0.04 µg/ml	Biolegend	400523	Flow cytometry
Rat IgG2b-AF647 iso ctrl	RTK4530	0.05 µg/ml	Biolegend	400626	Flow cytometry/ Imaging
Rat IgG2b-PE/Cy7 iso ctrl	RTK4530	0.04 µg/ml	Biolegend	400617	Flow cytometry
Rat IgG2b-APC/Cy7 iso ctrl	RTK4530	0.05 µg/ml	Biolegend	400623	Flow cytometry
Arm. Hamster-APC/Cy7 iso ctrl	HTK888	0.04 µg/ml	Biolegend	400927	Flow cytometry
CD11b-biotin	M1/70	0.05 µg/ml	BD	553309	Imaging
CD16/32 (labelled in-house)	2.4G2	0.05 µg/ml	BD	553142	Imaging
CD11c-BV605	N418	0.05 µg/ml	Biolegend	117333	Imaging
CD68-AF488	FA11	0.05 µg/ml	Biolegend	130712	Imaging
CD209b (labelled in-house)	eBio22D1	0.05 µg/ml	Invitrogen	14-2093-81	Imaging
Collagen I (labelled in-house)	COL-1	0.05 µg/µl	Invitrogen	PA-95137	Imaging
FDC-M1	FDC-M1	see methods	BD	551320	Cell enrichment
Rat IgG2c, κ iso ctrl	A23-1	see methods	BD	553982	Cell enrichment
Biotin anti-Rat Ig, κ	MRK-1	see methods	BD	553871	Cell enrichment
Goat anti-mouse κ IgG	polyclonal	see methods	SouthernBio	1050-01	IC
Donkey anti-goat IgG (H+L)	polyclonal	see methods	Jackson	705-005-147	IC
Talin-1	97H6	0.05 µg/ml	Abcam	ab108480	Imaging
Goat anti-mouse F(ab’)$_2$ AF647	polyclonal	0.002 µg/ml	Cell Signaling	4110	Imaging
NF-κB (p65)	D14E12	0.02 µg/ml	Cell Signaling	8242	Imaging
Goat anti-rabbit F(ab’)$_2$ AF647	polyclonal	0.002 µg/ml	Cell Signaling	4414	Imaging
CD45R/B220 BV421	RA3-6B2	0.02 µg/ml	BD	562922	Imaging
Arm. Hamster iso ctrl NP or NIP (labelled in-house)	HTK888	see methods	BioLegend	400901	IC
Goat anti-Arm. Hamster DyLight 649	polyclonal	see methods	BioLegend	405505	IC

Table S1 Antibodies used in this study.

Inhibitor	Target	Final conc.	Supplier	Cat. No.
Mycalolide B	Actin severing	3 μM	Santa Cruz	SC-358736
Latrunculin B	Actin severing	2 μM	Cayman Chemical	76343-94-7
Jasplakiolide (SiR-Actin)	Actin stabilisation	100 nM	Spirochrome	SC001:SiR-actin kit
CK-666	Arp2/3	100 μM	Sigma	SML0006
SMIFH2	Formin FH2 domain	20 μM	Merck	344092
Y-27632	Rho-associated protein kinase	1 μM	Cambridge Bioscience	SM02-1
(S)-nitro-blebbistatin	Non-muscle myosin II ATPase	100 μM	Cambridge Bioscience	CAY24171

Table S2 Inhibitors used in this study.

% Acrylamide	% Bis-Acrylamide	Young's modulus (kPa) (mean ± SEM)	No. Gels measured
4.96	0.05	1.88 ± 21.3	2
5.56	0.27	16.25 ± 0.77	2
7.06	0.20	24.24 ± 0.65	2
9.91	0.43	176.85 ± 0.21	2

Table S3 Polyacrylamide gel rigidities measured by atomic force microscopy (AFM).
